# Fermented Gold Kiwi for Improved Gastric Health: Evaluation of Efficacy and Safety in a Randomised, Double-Blind, Placebo-Controlled Trial

**DOI:** 10.3390/nu16162670

**Published:** 2024-08-13

**Authors:** Seon Mi Shin, Sang Jun Youn, Yong Choi, Bong Min Kim, Na Young Lee, Hyun Jeong Oh, Hyuck Se Kwon, Heung Ko

**Affiliations:** 1 Department of Internal Medicine, College of Korean Medicine, Semyung University, Semyeong-ro 65, Jecheon-si 27136, Republic of Korea; bunggujy21@hanmail.net; 2 RnBS Corporation, Seoul 06032, Republic of Korea; sjyoun@rnbs.co.kr (S.J.Y.); choiy@rnbs.co.kr (Y.C.); bmkim@rnbs.co.kr (B.M.K.); 3 R&D Team, Food and Supplement Health Claims, Vitech, Wanju 55365, Republic of Korea; lny4921@nate.com (N.Y.L.); hyunjeong2985@nate.com (H.J.O.); sek2gun@nate.com (H.S.K.)

**Keywords:** fermented gold kiwi, placebo-controlled study, randomised trial, nutritional supplement, gastrointestinal dysfunction, functional dyspepsia

## Abstract

This randomised double-blind placebo-controlled trial evaluated the efficacy and safety of fermented gold kiwi (FGK) in improving gastrointestinal health. A total of 100 participants were enrolled and randomly assigned to treatment or placebo groups. Over 8 weeks, the participants consumed an FGK or placebo preparation daily. Primary outcomes included changes in gastrointestinal symptoms assessed using the Gastrointestinal Symptom Rating Scale (GSRS) and the Korean version of the Nepean Dyspepsia Index (NDI-K), as well as quality of life assessed using the Functional Dyspepsia-related Quality of Life questionnaire. The FGK group showed significant improvements in GSRS and NDI-K total and subdomain scores compared with the placebo group. Moreover, the quality of life scores were significantly better in the FGK group than in the placebo group. Safety evaluations revealed no significant adverse events or clinically meaningful changes upon assessing laboratory test results. This study demonstrated that FGK is a safe and effective dietary supplement for improving gastrointestinal health in adults with gastrointestinal symptoms.

## 1. Introduction

According to statistics from the Korea Health Insurance Review and Assessment Service in 2021, gastrointestinal health-related diseases ranked high on the list of frequent diseases that resulted in outpatient visits and hospitalisations in all age groups, from teenagers to individuals in their 80s [1]. The prevalence of functional dyspepsia is reported to be 10–30% worldwide, whereas its prevalence in Korea is 8.1–37.0%. Moreover, atrophic gastritis, characterised by thinning of the gastric mucosa due to chronic inflammation and damage, is a common condition with a reported prevalence of approximately 40.7% in Korea; notably, a study on risk factors for functional dyspepsia in a Korean health examination population reported that 31.8% of patients with functional dyspepsia also had atrophic gastritis [2,3]. Globally, the prevalence of gastrointestinal diseases and functional dyspepsia is increasing, with significant regional differences. High-income regions such as South Korea have reported increased cases of gastric cancer and inflammatory bowel disease, whereas developing regions face higher burdens of infectious and chronic liver diseases. This trend underscores the need for implementing effective public health strategies and exploring the potential of natural functional foods in managing these conditions [4].

Considering the high prevalence, high disease burden, and large number of potential patients with functional dyspepsia and gastritis, effective symptomatic and therapeutic interventions are needed. The most commonly used medications are prokinetics and acid-suppressing drugs (proton pump inhibitors and H_2_-receptor blockers). However, based on the drug type and age group, long-term drug administration warrants caution due to possible adverse effects; therefore, a growing need exists for the development of functional food that can improve digestive function and protect the gastric mucosa without unwanted effects [5,6,7]. Kiwi is an *Actinidia* plant that contains various bioactive substances, including organic acids (quinic, malic, and citric acids), flavonoids, and carotenoids; importantly, their concentrations are 2–3 times higher than those in apples and grapes. Studies related to kiwi properties include analyses of the chemical composition, antioxidant activity, neuroprotective effect, inhibition of tyrosinase activity, antibacterial activity, inhibition of gastric cancer cell proliferation, meat tenderisation, and improvement of constipation [8,9]. Fermented gold kiwi (FGK) has been shown to improve the antioxidant and anti-inflammatory properties in vivo, protect the gastric mucosa by reducing inflammation, and increase prostaglandin E2 (PGE2) secretion in animal models of acute gastritis [9]. FGK has shown promise in preclinical studies as a dietary supplement to help improve gastric health; however, it has not been studied in humans [8,9]. Therefore, this study was conducted in Korea to assess the efficacy and safety of lactic acid bacteria FGK in improving gastrointestinal health in adults experiencing symptoms such as functional dyspepsia.

## 2. Materials and Methods

### 2.1. Study Design and Participants

**Figure 1:**
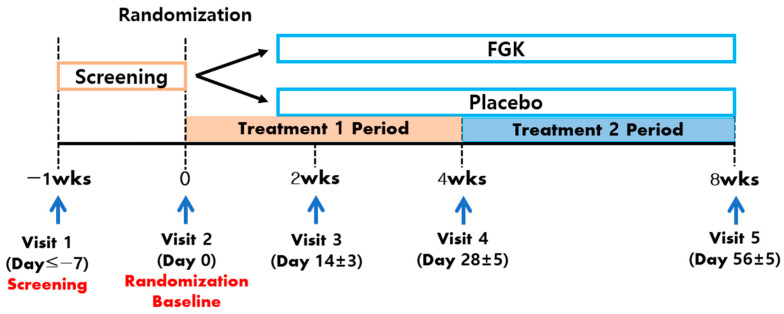
Study Flow. FGK, fermented gold kiwi.

This randomised, double-blind, placebo-controlled clinical trial enlisted adults experiencing gastrointestinal symptoms by posting a written notice on the hospital homepage and bulletin board of Semyung University Korean Medicine Hospital in Jecheon, Chungcheongbuk-do, Republic of Korea, until the target number of participants was achieved. Interested individuals visited the Department of Internal Medicine and were screened based on the participant selection criteria outlined in Table 1. The entire study duration for each participant was approximately 9 weeks, including a wash-out period of up to 7 days and a safety assessment conducted 2 weeks after the final visit. If participants had a history of using prohibited medications or foods (see Table S1), a wash-out period of up to 14 days was mandated (Figure 1). Participants were randomly allocated to either the treatment or placebo group during their second visit (V2), which took place within 1 week of the initial visit (V1), serving as the baseline. Before randomisation, the inclusion and exclusion criteria were re-evaluated, and eligible participants were enrolled. Baseline assessments were conducted, and participants received supplies for the 33-day study period (refer to Section 2.2. Intervention). Follow-up visits were scheduled for 14 ± 3 days (V3), 28 ± 5 days (V4), and 56 ± 5 days (V5) after the baseline visit (V2). During visits V3 through V5, vital signs, medical history, and concomitant medication use were reviewed, and efficacy and safety were assessed. Laboratory and pregnancy tests were performed during visits V1 and V5. Participants were reminded of their visit schedules by the clinical trial investigator.

### 2.2. Intervention

**Figure 2:**
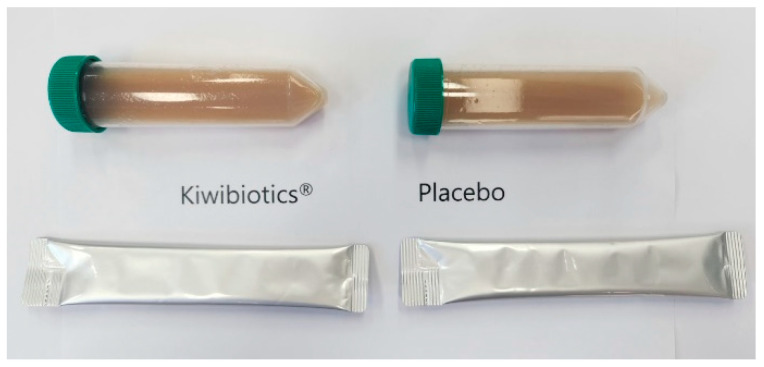
Intervention and placebo preparations.

Based on a previous study [9], the daily FGK intake was set as outlined in Table S2. The intervention (two packets of 5 mL each, the daily dose is 10 mL.) or placebo (same appearance and volume) preparation was consumed orally once daily for 8 weeks (Figure 2). For Kiwibiotics^®^ production, gold kiwi (*Actinidia chinensis* L.) puree without peels and seeds was purchased from Namuang Foods. Vitech. Co., Ltd.( Wanju, Republic of Korea) Bacteria used for fermentation included *Lactococcus lactis VI-01* (KTCT 14351 BP), *Lacticaseibacillus paracasei VI-02* (KTCT 14352 BP) isolated from gold kiwi, *Lacticaseibacillus casei VIGRA01* (KTCT 14756 BP), *Lactobacillus helveticus VICAM05* (KTCT 15949 BP) isolated from cheese, and *Lactobacillus acidophilus* VIFEC24 (KTCT 15950 BP) isolated from infant faeces; each strain was cultured in MRS Broth at 37 °C. Collected seed-cultured bacteria were pre-cultured for 12 h at 37 °C in an industrial medium. The pre-culture solution was mixed with the prepared kiwi puree and cultured at 37 °C for 8 h to prepare FGK.

### 2.3. Randomisation and Blinding

At V2, participants were allocated to either the placebo or the experimental group using stratified block randomisation based on sex, ensuring a 1:1 ratio. This method was employed to minimise allocation bias, enhance group comparability, and achieve balanced distribution. A random number sequence starting with participant number 1 was generated using the SAS^®^ system’s randomisation programme. During the preparation of the intervention, labels were attached according to the IP code list, and the labelled interventions were provided to the test institution before the trial began.

The randomisation process was conducted through a web-based interactive web response system (IWRS), which allowed for reproducibility using a designated seed. The IWRS is a centralised randomisation system used in clinical trials, providing real-time web-based randomisation to ensure consistency and integrity of research data. Researchers use a user-friendly web interface to randomise study participants in real-time, managing data in a secure environment. It allows customisation of various randomisation algorithms to meet diverse research requirements. The IP manager or pharmacist was responsible for supplying the intervention with an IP number assigned to each participant. In instances of defect or damage to the intervention, a new IP number was assigned via the IWRS to maintain consistency in the treatment group.

The randomisation code and IP number were handled by a third-party individual who was not blinded to the data. To preserve the double-blind nature of the study, details regarding participant allocation, serious adverse reactions, and codes related to the production, packaging, and labelling of the trial products were sealed in an envelope by the trial supervisor. The code was kept confidential until the study’s conclusion, except in situations where it was imperative to check the code.

### 2.4. Endpoints

The primary functional outcome variables were, first, the change in the Gastrointestinal Symptom Rating Scale (GSRS) upper gastrointestinal symptoms total score from baseline (V1) to week 8 (V5), and second, the change in the Korean version of the Nepean Dyspepsia Index (NDI-K) score from baseline to week 8 (V5). The GSRS is a self-reported gastrointestinal symptom scale that includes both upper and lower gastrointestinal symptoms with a total of 15 symptom items, and each symptom can be scored on a 7-point scale. Upper gastrointestinal symptoms include eight items: abdominal pain, heartburn, acid regurgitation, pain that is alleviated by eating or taking antacids on an empty stomach, nausea, borborygmus, abdominal distension, and eructation. Lower gastrointestinal symptoms include seven items: increased flatus, decreased passage of stools, increased passage of stools, loose stools, hard stools, urgent need for defecation, and feeling of incomplete evacuation [10,11,12,13]. The NDI-K is a questionnaire that measures functional dyspepsia symptoms and quality of life. The symptom scorecard of the NDI-K is based on 15 symptoms: upper abdominal pain, upper abdominal discomfort, upper abdominal heartburn, heartburn, upper abdominal cramps, chest pain, early satiety, acid reflux, postprandial fullness, upper abdominal pressure, upper abdominal bloating, nausea, eructation, vomiting, and shortness of breath. Each item is rated for frequency, intensity, and distress. Frequency is rated on a 5-point scale based on how many days the symptoms occurred in the past 2 weeks (0: never, 1: 1–4 days, 2: 5–8 days, 3: 9–12 days, 4: every or almost every day). The intensity of symptoms is rated on a 6-point scale (0: not at all, 1: very mild, 2: mildly present, 3: mildly present, 4: severe, 5: very severe), and the severity of distress is rated on a 5-point scale (0: not at all, 1: very mild, 2: mild. 3: severe, 4: very severe). Symptom severity was assessed by calculating the total score of symptom frequency, intensity, and distress [14,15]. The NDI-K includes eight symptoms specific to dyspepsia: epigastric pain (aching or stiffness), epigastric discomfort (not painful or burning), epigastric distress (heartburn, burning), early satiety (inability to eat a normal-sized meal), postprandial fullness (feeling full, gassy, or indigestion), epigastric pressure, epigastric bloating, and nausea [16,17]. The values measured at V1 were used as baseline values. One study evaluated the efficacy of mosapride and rabeprazole in treating functional dyspepsia using the NDI-K involving 30 patients, with 27 completing the final analysis [18]. Another study investigated the effects of electrolysed alkaline-reduced water on functional dyspepsia symptoms, using GSRS and NDI-K scores, and found significant improvements in both metrics [19].

The secondary functional endpoints were as follows. First, the changes in the GSRS upper gastrointestinal symptoms total score from baseline to V3 and V4. Second, the change in the NDI-K score from baseline to weeks 2 and 4 to determine whether dyspepsia symptoms changed in the short term. Third, the change in the GSRS total score, lower gastrointestinal symptoms total score, total score for each categorised symptom (reflux, abdominal pain, dyspepsia, diarrhoea, constipation), and the score of each symptom subscale (each question) from baseline to weeks 2, 4, and 8. This provides an overall assessment of the various symptoms of the gastrointestinal tract and the specific changes for each symptom. Fourth, the change in the Functional Dyspepsia-related Quality of Life scale (FD-QoL) scores from baseline to weeks 2, 4, and 8. The FD-QoL, a 21-item questionnaire with four subdomains (eating domain, life vitality, emotional domain, and social functioning), has been used to assess the quality of life related to dyspepsia. Each question is answered on a 5-point Likert scale (0: not at all, 1: a little bit, 2: moderately, 3: a lot, and 4: very much) [20,21]. Finally, the changes in tumour necrosis factor (TNF)-α and interleukin (IL)-6 concentrations from baseline to week 8 were analysed using enzyme-linked immunosorbent assay.

Lifestyle questionnaires were administered at each visit and included questions about smoking, dietary habits, perceived stress, alcohol consumption, exercise habits, and caffeine intake. Dietary questionnaires were completed at V2–V5 using a 24-h recall method to describe the diet of the previous day. The 24-h recall dietary diary reflects the approximate food and nutrient intake that the participant had consumed on the day before the visit, and the total calorie (kcal) intake was analysed using the CAN-PRO program 5.0 (Computer Aided Nutritional Analysis Program for Professionals) developed by the Korean Nutrition Society. At each visit, participants were advised to maintain their usual dietary patterns, physical activity, and dietary intake and to avoid the regular concomitant consumption of any contraindicated foods. During the study, participants were instructed to maintain their lifestyle as closely as possible to that on visit 1. Exclusion criteria were implemented to exclude excessive alcohol consumption, and participants were instructed at each visit to avoid excessive alcohol consumption and overeating and to maintain a regular diet and exercise regime.

### 2.5. Safety

Adverse reactions and unwanted effects were evaluated through interviews during the visit and through blood and urine tests at V1 and V5.

### 2.6. Sample Size Calculation

Calculation of the number of study participants was based on the results of Choi et al. [10], who reported a mean ± standard deviation (SD) for the change in the GSRS upper abdominal symptoms score of −4.4 ± 3.8 (*n* = 38) in the test group and −2.1 ± 3.4 (*n* = 35) in the control group. Using these results, the effect size was assumed to be −2.3 and joint SD to be 3.6. Based on the change in the GSRS epigastric symptoms score, the number of participants required to achieve a 5% significance level and 81% power was calculated to be 40 participants per arm. To account for a 20% dropout rate, the enrolment of 50 participants per arm was planned, accounting for a total of 100 participants. The calculation of the number of study participants was performed using SAS^®^ version 9.4 (SAS Institute, Cary, NC, USA).

### 2.7. Statistical Analyses

The significance level for statistical analyses was set at 5%, and two-tailed tests were performed. Continuous variables are presented as the number of participants, mean, and SD, and categorical variables are presented as frequencies and percentages. For continuous variables, the unpaired *t*-test or Wilcoxon’s rank-sum test was performed for between-group comparisons, and the paired *t*-test or Wilcoxon’s signed-rank test was used for within-group comparisons, depending on normality. For categorical variables, between-group comparisons were performed using Pearson’s chi-square test or Fisher’s exact test, and within-group comparisons were performed using McNemar’s test. The analysis was conducted by selecting either the chi-square test or Fisher’s exact test, depending on the assumptions of the statistical analysis. If more than 20% of the cells have expected counts below 5, then the chi-square test may not be valid. In this case, we performed Fisher’s exact test. Demographic, medication, and medical history data are presented for the Per Protocol Set (PPS) analysis arm.

The safety population comprised individuals who were enrolled in this trial, received the investigational food product, and had at least one safety event for which safety information was collected. The Full Analysis Set comprised all participants who were randomised to receive the investigational product and underwent at least one functional assessment after consuming the investigational product in accordance with the intention-to-treat principle. The PPS of the functional evaluation group was composed of participants who completed this trial, had no major violations affecting the results of this trial (e.g., exclusion criteria and major protocol violations), and had a compliance rate of 80% or higher.

For each endpoint, the mean, SD, median, minimum, and maximum of the baseline (V1) score, the score at week 8 (V5), and the percent change from V1 to V5 are presented for each arm. Comparisons between groups were analysed by fitting analysis of covariance (ANCOVA) with the change in score from V1 to V5 as the response variable and the score at V1 as a covariate. If additional factors affecting the outcome variable were identified, a general linear model was fitted, which is an extension of the ANCOVA model by adding these factors as covariates. When the parametric methods were considered inappropriate owing to data bias, the nonparametric Wilcoxon’s rank-sum test was applied.

Comparisons of changes in GSRS epigastric symptom scores between the two study groups over time (V1, V3, V4, and V5) were analysed using repeated-measures analysis of variance followed by a test of interaction between the two groups over time.

All adverse reactions were standardised into ‘System Organ Class’ and ‘Preferred Term’ using MedDRA^®^ (Medical Dictionary for Regulatory Activities) Version 25.0. MedDRA^®^ is a standardisation scheme established by the Council for Harmonisation and on the technical requirements for human use of medicinal products. In addition, Common Terminology Criteria for Adverse Events Version 5.0 was used to compare the degrees of symptoms of adverse reactions, evaluate their associations with the test drug, and to determine which participants experienced an adverse reaction, adverse drug reaction, or serious adverse reaction at least once during the clinical trial period. For participants, the number of individuals (appearance rate) and the number of cases were calculated. In addition, the 95% confidence interval for the expression rate was determined, and the statistical significance of the expression rate between the administration groups was examined using the chi-square or Fisher’s exact test. All statistical analyses were performed using SAS^®^ version 9.4.

## 3. Results

### 3.1. Participant Characteristics

**Figure 3:**
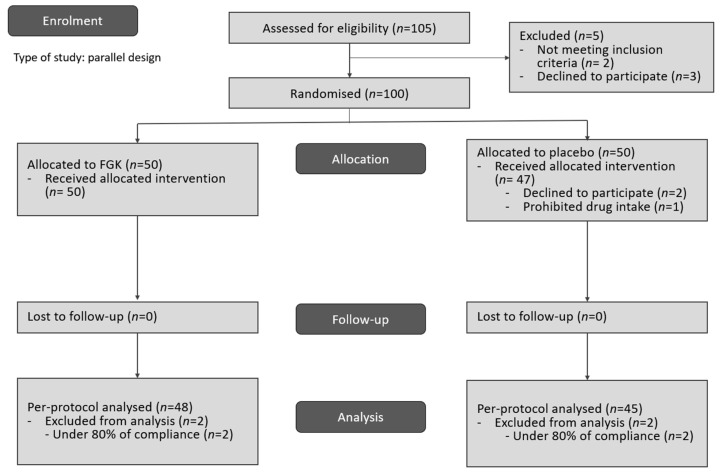
Trial flowchart. FGK, fermented gold kiwi.

The participants were screened between 9 March 2023 and 27 January 2024. To select eligible participants, 105 candidates were screened. Of these, five participants were excluded, resulting in 100 participants who were randomised into the FGK and placebo groups (*n* = 50 each). Three participants of the placebo group dropped out, leaving 97 participants who completed the study (FGK group: *n* = 50, placebo group: *n* = 47; Figure 3, Table 2).

### 3.2. Study Endpoints

**Figure 4:**
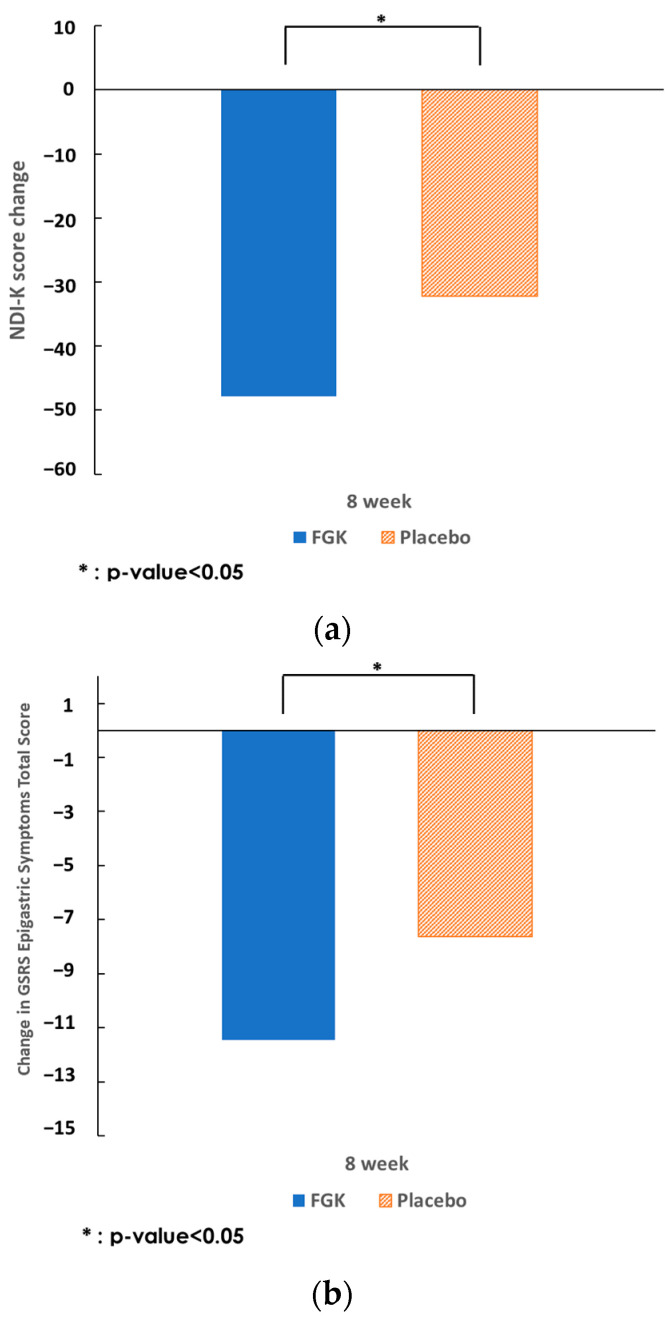
Changes in the GSRS epigastric symptoms (**a**) and NDK-I score (**b**) at 8 weeks from baseline (Per Protocol Set). FGK, fermented gold kiwi; GSRS, Gastrointestinal Symptom Rating Scale; NDI-K, Korean version of the Nepean Dyspepsia Index.

The primary endpoints, changes in GSRS epigastric symptom scores, and NDI-K score from baseline to week 8 were as follows. Before consumption (V1), the GSRS epigastric symptoms scores did not significantly differ between the FGK (25.10 ± 7.01) and placebo (26.13 ± 7.13) groups. After 8 weeks (V5), the GSRS epigastric symptoms scores were significantly lower in the FGK group (13.65 ± 6.70) than in the placebo group (18.49 ± 9.66, *p* = 0.0052). Compared to V1, both groups showed significant decreases (FGK: −11.46 ± 7.89, *p* < 0.0001; placebo: −7.64 ± 8.66, *p* < 0.0001). The change in GSRS epigastric symptoms scores after 8 weeks was significantly larger in the FGK group than in the placebo group (*p* = 0.0069). Repeated-measures analysis of variance comparing the change in GSRS epigastric symptoms scores between the two dietary groups over time confirmed an interaction of dietary group and time of visit (*p* = 0.0083; Table 3, Figure 4).

The NDI-K score before intervention (V1) was not significantly different between the two study groups (FGK: 72.92 ± 22.85, placebo: 72.51 ± 24.67); however, the NDI-K score after 8 weeks (V5) was significantly lower in the FGK group (25.06 ± 22.63) than in the placebo group (40.36 ± 34.17, *p* = 0.0364). Compared to baseline, both groups showed significant decreases after 8 weeks (FGK: −47.85 ± 29.77, *p* < 0.0001; placebo: −32.16 ± 30.88, *p* < 0.0001). At 8 weeks, the change in NDI-K score was significantly larger in the FGK group than in the placebo group (*p* = 0.0073). The repeated-measures analysis of variance comparing the change in NDI-K scores between the two groups over time confirmed an interaction between group and time of visit (*p* = 0.0061; Table 3).

The results of the secondary endpoints were as follows. The GSRS upper gastrointestinal symptoms total scores at baseline (V1) were 25.10 ± 7.01 in the FGK group and 26.13 ± 7.13 in the placebo group, with no difference between the two groups. The GSRS upper gastrointestinal symptoms total scores at 2 weeks (V3) were 18.27 ± 5.52 in the FGK group and 19.31 ± 6.64 in the placebo group, with no difference between the study groups. Compared to baseline, both groups showed significant decreases (FGK: −6.83 ± 6.25, *p* < 0.0001; placebo: −6.82 ± 5.75, *p* < 0.0001), but the change in GSRS upper gastrointestinal symptoms total score did not significantly differ between the two study groups after 2 weeks (Table S3). After 4 weeks (V4), the GSRS upper gastrointestinal symptoms total score was significantly lower in the FGK group (15.21 ± 4.96) than in the placebo group (18.96 ± 8.08, *p* = 0.0335). Compared to baseline, both groups showed significantly decreased scores at 4 weeks (FGK: −9.90 ± 6.90, *p* < 0.0001; placebo: −7.18 ± 6.85, *p* < 0.0001); however, the score change was significantly larger in the FGK group than in the placebo group (*p* = 0.0082; Table S3).

Regarding changes in NDI-K scores, the NDI-K score did not significantly differ between the two groups either at baseline (V1; FGK: 72.92 ± 22.85, placebo: 72.51 ± 24.67) or after 2 weeks (V3; FGK: 43.81 ± 24.44, placebo: 47.82 ± 26.51). Compared to baseline, both FGK (−29.10 ± 20.04, *p* < 0.0001) and placebo (−24.69 ± 23.30, *p* < 0.0001) groups showed significant decreases; however, these changes in the NDI-K score after 2 weeks were not significantly different between the two groups (Table S3). At 4 weeks (V4), the NDI-K score was 33.75 ± 22.12 in the FGK group and 44.27 ± 30.58 in the placebo group, with no difference between the two groups. Compared to baseline, both FGK (−39.17 ± 21.20, *p* < 0.0001) and placebo (−28.24 ± 25.16, *p* < 0.0001) groups showed significant decreases; the change in the NDI-K score after 4 weeks of treatment was significantly larger in the FGK group than in the placebo group (*p* = 0.0056; Table S3).

Regarding changes in the GSRS total score, the scores in the FGK group were 46.90 ± 12.16, 34.83 ± 9.96, 28.92 ± 8.37, and 25.98 ± 12.64 before (V1), and after 2 weeks (V3), 4 weeks (V4), and 8 weeks (V5), respectively. Significant decreases started at 2 weeks of FGK consumption and after 8 weeks of treatment; the GSRS total score changed by −20.92 ± 14.15. In the placebo group, the GSRS total scores were 48.53 ± 11.90, 36.04 ± 12.82, 34.18 ± 13.47, 34.09 ± 17.12, and 34.09 ± 17.12 at V1, V3, V4, and V5, respectively. Significant decreases started at 2 weeks of placebo consumption, and the score change after 8 weeks was −14.44 ± 15.04. The change in the GSRS total score after 8 weeks (V5) was significantly greater in the FGK group than in the placebo group (*p* = 0.0121; Table S4).

Regarding the GSRS total scores of lower gastrointestinal symptoms, the FGK group had scores of 21.79 ± 7.10, 16.56 ± 5.59, 13.71 ± 4.31, and 12.33 ± 6.23 at V1, V3, V4, and V5, respectively. Significant decreases started at 2 weeks of FGK consumption; the score change after 8 weeks was −9.46 ± 7.72. In the placebo group, the lower gastrointestinal symptoms total scores were 22.40 ± 6.94, 16.73 ± 7.11, 15.22 ± 6.57, and 15.60 ± 8.20 at V1, V3, V4, and V5, respectively. Significant decreases started at 2 weeks, and the score change after 8 weeks was −6.80 ± 8.19. The change in the total score of lower gastrointestinal symptoms after 8 weeks was significantly greater in the FGK group than in the placebo group (*p* = 0.0333; Table S4).

Furthermore, changes in the scores of individual symptoms (reflux, abdominal pain, dyspepsia, diarrhoea, and constipation) assessed in the GSRS were analysed. Of these symptoms, only reflux and diarrhoea were significantly different between the two treatment groups after 8 weeks of consumption.

In the FGK group, the total scores of reflux symptoms were 3.04 ± 1.25, 2.13 ± 0.90, 1.69 ± 0.58, and 1.45 ± 0.84 at V1, V3, V4, and V5, respectively. Significant decreases started at 2 weeks, and the score change after 8 weeks was −1.59 ± 1.31. In the placebo group, the total scores of reflux symptoms were 2.97 ± 1.21, 2.12 ± 1.02, 2.21 ± 1.11, and 2.17 ± 1.35 at V1, V3, V4, and V5, respectively. Significant decreases started at 2 weeks, but the scores increased again at 4 weeks, and the score change after 8 weeks was −0.80 ± 1.07. The changes in the total score of reflux symptoms at V4 and V5 were significantly larger in the FGK group than those in the placebo group (V4: *p* = 0.0235, V5: 0.0045; Table S5).

The total scores of diarrhoea symptoms in the FGK group were 2.82 ± 1.14, 2.26 ± 0.97, 1.78 ± 0.76, and 1.58 ± 0.94 at V1, V3, V4, and V5, respectively. Significant decreases started at 2 weeks of FGK consumption with a change of −1.24 ± 1.05 after 8 weeks of FGK consumption. In the placebo group, the total scores of diarrhoea symptoms were 2.82 ± 1.16, 2.19 ± 1.10, 1.91 ± 0.87, and 2.04 ± 1.24 at V1, V3, V4, and V5, respectively. Significant decreases were observed from 2 weeks of intervention. However, the scores increased again at 8 weeks; the change after 8 weeks was −0.78 ± 1.19. The change in the total score of diarrhoea symptoms at 8 weeks (V5) was significantly larger in the FGK group than that in the placebo group (*p* = 0.0240; Table S5).

The total scores of abdominal pain, dyspepsia, and constipation symptoms in the GSRS continued to decrease from 2 weeks to 8 weeks of intervention, whereas in the placebo group, only the total score of abdominal pain symptoms decreased from 2 weeks to 8 weeks. In the placebo group, total scores of dyspepsia and constipation decreased at 2 weeks and remained unchanged or increased at 4 weeks (Tables S6 and S7).

Moreover, the changes in FD-QoL scores were analysed. The FD-QoL scores in the FGK group were 21.17 ± 10.34, 14.69 ± 10.17, 11.00 ± 9.60, and 8.02 ± 10.11 at V2, V3, V4, and V5, respectively. Significant decreases started at 2 weeks of FGK consumption, and the score change after 8 weeks was −13.15 ± 13.34 (Table S8). In the placebo group, the FD-QoL scores were 19.67 ± 11.65, 16.58 ± 11.83, 15.62 ± 12.39, and 14.47 ± 14.58 at V2, V3, V4, and V5, respectively. Significant decreases started at 2 weeks, and the score change after 8 weeks was −5.20 ± 8.47. The changes in FD-QoL scores after 4 (V4) and 8 (V5) weeks of intervention were significantly greater in the FGK group than in the placebo group (V4: *p* = 0.0024, V5: *p* < 0.0001; Table S8).

The FD-QoL questionnaire analyses changes in the subdomains of the eating, life vitality, emotional, and social functioning domains.

In the FGK group, the FD-QoL eating domain scores were 5.81 ± 3.13, 4.17 ± 3.40, 2.98 ± 3.28, and 1.81 ± 2.94 at V2, V3, V4, and V5, respectively. Significant decreases in FD-QoL scores started at 2 weeks of treatment, with a change of −4.00 ± 4.19 after 8 weeks of treatment. In the placebo group, the FD-QoL eating domain scores were 5.18 ± 3.16, 4.42 ± 3.14, 4.11 ± 3.28, and 3.80 ± 4.20 at V2, V3, V4, and V5, respectively. Significant decreases started at 4 weeks of intervention, and the score change after 8 weeks was −1.38 ± 2.87. The changes in FD-QoL eating domain scores at 4 (V4) and 8 (V5) weeks of intervention were significantly greater in the FGK group than in the placebo group (V4: *p* = 0.0018, V5: *p* < 0.0001; Table S9).

The scores in the FD-QoL life vitality domain in the FGK group were 6.79 ± 3.24, 4.92 ± 2.91, 4.10 ± 2.86, and 3.42 ± 3.46 at V2, V3, V4, and V5, respectively. Significant decrease in the score started at 2 weeks of intervention, with a change of −3.38 ± 3.16 after 8 weeks. In the placebo group, FD-QoL life vitality domain scores were 7.00 ± 3.38, 6.00 ± 3.57, 5.51 ± 3.86, and 4.89 ± 3.91 at V2, V3, V4, and V5, respectively. Significant decreases started at 2 weeks of intervention, with a change of −2.11 ± 3.44 after 8 weeks (Table S9). Significantly larger reductions in FD-QoL life vitality domain scores were found at 4 (V4) and 8 (V5) weeks in the FGK group compared to those in the placebo group (V4: *p* = 0.0375, V5: *p* = 0.0379; Table S9).

In the FGK group, the FD-QoL emotional domain scores were 4.79 ± 3.74, 2.85 ± 2.77, 1.94 ± 2.68, and 1.52 ± 2.52 at V2, V3, V4, and V5, respectively. Significant decreases in FD-QoL scores started at 2 weeks of intervention, with a change of −3.27 ± 4.28 after 8 weeks (Table S10). In the placebo group, FD-QoL emotional domain scores were 3.98 ± 3.89, 3.42 ± 3.82, 3.36 ± 3.69, and 3.07 ± 4.31 at V2, V3, V4, and V5, respectively. Significant decreases were observed after 8 weeks of intervention, with a change of −0.91 ± 2.61 (Table S10). Significantly greater reductions in FD-QoL emotional domain scores were observed at 2 (V3), 4 (V4), and 8 (V5) weeks in the FGK group compared to those in the placebo group (V3: *p* = 0.0465, V4: *p* = 0.0006, V5: *p* = 0.0005; Table S9).

The FD-QoL social functioning scores in the FGK group were 3.77 ± 3.46, 2.75 ± 2.99, 1.98 ± 3.00, and 0.27 ± 2.34 at V2, V3, V4, and V5, respectively. Significant decreases in FD-QoL scores started at 2 weeks of intervention, with a change of −2.50 ± 3.76 after 8 weeks. In the placebo group, the FD-QoL social functioning scores were 3.51 ± 4.08, 2.73 ± 3.73, 2.64 ± 3.65, and 2.71 ± 4.62 at V2, V3, V4, and V5, respectively. Significant decreases started at 2 weeks of intervention, and the score change after 8 weeks was −0.80 ± 2.62. The change in FD-QoL social functioning score at 8 weeks (V5) was significantly greater in the FGK group than in the placebo group (*p* = 0.0035; Table S10).

The changes in TNF-α and IL-6 concentrations from baseline to week 8 were not significantly different in either treatment group (Table S11).

### 3.3. Safety

The safety analysis included 50 clinical trial participants in the FGK group and 48 participants in the placebo group who had consumed at least one dose of the assigned trial intervention.

A total of 10 adverse events occurred in 8 participants (16.00%) of the FGK group, and 17 adverse events occurred in 10 participants (20.83%) of the placebo group, with no significant difference between the groups (*p* = 0.5368, chi-square test). No adverse drug reactions or serious adverse events were associated in the FGK and placebo groups (Table S12).

The adverse events in the FGK group most commonly included pharyngitis (*n* = 3) and rhinitis (*n* = 3), followed by dysmenorrhoea, headache, motion sickness, and pruritus (*n* = 1 each). The adverse reactions in the placebo group included dysmenorrhoea (*n* = 5), followed by headache (*n* = 4), pharyngitis (*n* = 3), rhinorrhoea (*n* = 2), eye movement, dyspepsia, and temperature sensation (*n* = 1 each). All adverse reactions were mild and unrelated to FGK and placebo.

Among the blood chemistry items, the glucose levels significantly decreased by 1.56 ± 10.82 g/dL in the FGK group at 8 weeks (*p* = 0.0166) but did not show a significant difference in the placebo group. However, all changes were within the normal range and not clinically significant (Table S13). None of the other test items showed statistically or clinically significant changes. Regarding protein in the urine, nine participants in the FGK group had normal values after the intervention out of 10 participants who had abnormal values at baseline. This change from pre- to post-intervention values was significant (*p* = 0.0348; Tables S14 and S15). Moreover, vital signs showed no significant differences within and between the groups (Table S16).

### 3.4. Physical Activity and Diet

Analyses of lifestyle habits demonstrated no significant differences between the two study groups regarding smoking status, dietary habits, perceived stress, exercise habits, alcohol consumption, caffeine intake, and dietary questionnaires at baseline (V2) and 8 weeks of intervention (V5; Tables S17 and S18).

## 4. Discussion

This study analysed the effects of FGK by assessing the upper and lower gastrointestinal symptoms, functional dyspepsia symptoms, and quality of life. The GSRS total score analysis revealed that the FGK group showed a significant reduction in symptoms starting at 2 weeks of intervention and demonstrated greater improvement than the placebo group at 4 and 8 weeks. In the NDI-K total score analysis, the FGK group showed a significant decrease after 2 weeks and a greater improvement than the placebo group after 8 weeks. In addition, the FD-QoL questionnaire results showed a significant improvement in quality of life in the FGK group after 2 weeks and a greater improvement than in the placebo group after 4 and 8 weeks. The safety evaluation demonstrated no significant differences between the two treatment groups and no clinically meaningful changes in laboratory test values, confirming the safety of FGK.

Although the precise cause of gastritis remains unclear, ethanol-induced formation of reactive oxygen species (ROS) reportedly contributes to its development [22,23]. Excessive ROS production leads to oxidative stress, a factor implicated in many diseases [24,25]. Normally, small amounts of ROS generated in the stomach are rapidly neutralised by various antioxidant enzymes and other antioxidants. However, when ROS production is excessive, it can surpass the stomach’s antioxidant defence mechanisms, leading to oxidative stress and subsequent damage to the gastric mucosa [26,27]. Ulcer treatment heavily relies on the scavenging of free radicals [28]. Kiwi fruit is rich in antioxidants such as ascorbic acid, carotenoids, lutein, and flavonoids, which help mitigate oxidative stress by scavenging free radicals [29]. Previous studies [9,30] have shown that FGK has significant free radical scavenging activity, suggesting that FGKs may protect tissues from oxidative damage.

Based on previous findings on the anti-inflammatory and antioxidant properties of kiwi fruits [8,9], an experimental study was conducted to evaluate the inflammatory changes induced by FGK pretreatment in an animal model of chloral hydrate/ethanol-induced acute gastritis. FGK has been shown to have gastroprotective effects through several biochemical pathways. A study on its effects in an HCl/EtOH-induced gastric injury model in rats demonstrated that FGK inhibits inflammatory factors, such as iNOS, COX-2, IL-6, and TNF-α, while increasing the expression of the protective molecule PGE2. Additionally, FGK administration improved gastric lesions, reduced gastric fluid volume, and decreased free acidity, total acidity, and pepsin activity, indicating its potential in preventing and treating gastritis and gastric ulcers [9]. This mechanism is believed to result in symptom relief in the upper gastrointestinal tract.

PGE2 is crucial in maintaining gastric homeostasis by regulating blood flow and epithelial cell movement in the gastric mucosa and inhibiting the secretion of gastric mucous and acid [31]. Ethanol reduces mucosal PGE levels, with PGE2 being the most prevalent prostaglandin in the gastrointestinal tract [32]. Consequently, PGE2 is frequently used as a biomarker to assess the protective effects of various substances against gastritis [33,34]. Experimental studies have demonstrated that FGK exerts gastroprotective effects by enhancing PGE2 production in the gastric mucosa, indicating that PGE2 partially mediates FGK’s protective actions. Prostaglandins contribute to mucosal protection by stimulating mucous and bicarbonate secretion, maintaining mucosal blood flow, increasing epithelial cell resistance to injury, and reducing leukocyte recruitment [33]. These findings suggest that FGK aids in protecting the gastric mucosa by inhibiting acid secretion; moreover, clinical studies have demonstrated improvements in gastric function.

In patients with functional dyspepsia, placebo response rates range from 37.2% to 42.2%, according to existing studies [34,35]. To overcome this placebo effect, the study employed double-blinding and participant education, suggesting that participant education was conducted at each visit concerning the placebo effect and encouraging them to manage their expectations, which could reduce placebo response [36].

This study has several limitations. First, the single-centre design limits the generalisability of the results. When a study is conducted at a single centre, the recruitment or management of study participants may differ from other centres. This affects the external validity of the study results and can make it challenging to generalise the results to other regions or populations. For example, if patient characteristics or healthcare settings in a particular centre differ from those elsewhere, the results of that study may not be reproducible in other settings. Therefore, future studies should be multi-centre studies to reflect different populations and healthcare settings. Second, the short study period of 8 weeks may not be sufficient to assess long-term effectiveness or persistence. Longer follow-up periods are needed to determine the long-term effects of a treatment or intervention. Short-term studies can be useful for assessing the initial response; however, the possibility of diminishing effectiveness or side effects over time cannot be ruled out. Therefore, additional long-term follow-up studies are needed to assess long-term safety and efficacy. Third, the use of self-report questionnaires carries the potential for bias. These questionnaires allow research participants to subjectively assess their symptoms or condition, which may reduce the accuracy of results owing to issues, such as social desirability bias and recall bias. Study participants may underestimate or overestimate their condition, which can reduce the reliability of the results. To reduce this bias, the use of objective measures or biomarkers is preferred. Fourth, financial limitations did not allow us to perform specific metabolite and metabolome analyses. Metabolite analyses play an important role in understanding disease mechanisms and assessing treatment response. For example, metabolomic analyses can identify changes in specific metabolic pathways, which can help predict treatment effectiveness or side effects. Future studies should be well-funded to include such analyses. Fifth, further research is warranted on the modulation of symptom intensity and dose by sex and age. Symptom intensity and dose response may differ by sex and age, warranting further research. Sex and age are important factors that influence drug metabolism and response and are important for developing personalised treatment strategies; therefore, future studies should conduct more detailed analyses that consider these factors. Nevertheless, the randomised, double-blind, placebo-controlled design of this study increased the reliability of the study, and the comprehensive assessment of upper and lower gastrointestinal symptoms, functional dyspepsia symptoms, and quality of life allowed for a multifaceted analysis. These strengths contributed to the usefulness of the study results.

According to clinical trial results, FGK has been proven to have beneficial effects on stomach health. Participants experienced positive changes, indicating its potential as a stomach health supplement. As it is composed of natural ingredients, it is expected to be a safe long-term supplement with minimal side effects. Future prospects include the need for larger clinical trials stratified by symptom severity, age, and sex to further validate the efficacy and safety of gold kiwi extract. Additionally, developing various product forms for increasing consumer accessibility is crucial, which will help in establishing gold kiwi extract as a natural solution for improving stomach health. Researchers aim to identify the active components and study their mechanisms of action in more detail, thereby providing more efficient usage methods.

## 5. Conclusions

In this study, we assessed the efficacy and safety of FGK in improving the gastrointestinal health in adults. We evaluated the effects of FGK on the upper and lower gastrointestinal symptoms using the GSRS, on functional dyspepsia symptoms using the NDI-K, and on quality of life using the FD-QoL questionnaire. Furthermore, the safety of FGK administration was assessed after 8 weeks of intervention. Our data confirm that FGK is a safe ingredient that can enhance gastric health. Our findings are significant as they support FGK as a new treatment option for improving functional dyspepsia and gastrointestinal health.

## Figures and Tables

**Table 1:** Participant selection criteria.

**Inclusion**
Adult males and females aged 19–70 years
Participants with at least one of the Rome IV classified dyspepsia symptoms * and not requiring urgent pharmacologic treatment (onset of symptoms more than 6 months prior to V1, and symptoms present in the past 3 months) * Dyspeptic symptoms classified as Rome IV - Unpleasant postprandial fullness (bothersome postprandial fullness) - Unpleasant early satiation (bothersome early satiation) - Unpleasant epigastric pain (bothersome epigastric pain) - Unpleasant epigastric burning (bothersome epigastric burning)
Participants with at least two of the eight major symptoms of the NDI-K (epigastric pain, epigastric discomfort, epigastric burning, early satiety, postprandial fullness, epigastric pressure, epigastric bloating, and nausea) scoring moderate (3) or higher on at least two of the eight items and a total score of 6 or higher
Participants who agree to participate in this study and have voluntarily signed a written informed consent form.
**Exclusion**
History of peptic ulcer and reflux oesophagitis within 6 months prior to V1, individuals with a history of gastrointestinal surgery (except appendectomy and haemorrhoidectomy), and patients with a history of digestive system malignancy.
Participants who had taken H2 receptor blockers, anticholinergics (muscarinic receptor antagonists), gastrin receptor antagonists, prostaglandin preparations, proton pump inhibitors, gastric mucosal protectants, other drugs intended to treat gastritis, or gastric health supplements within 2 weeks prior to V1.
Participants who required continuous use of medications that may cause gastritis during the study, such as corticosteroids, non-steroidal anti-inflammatory drugs, or aspirin,
Participants with severe cerebrovascular disease (such as cerebral infarction and cerebral haemorrhage), cardiac disease (angina pectoris, myocardial infarction, heart failure, arrhythmia requiring treatment), or malignancy within 6 months prior to V1; however, patients with a history of cerebrovascular disease or cardiac disease but who are clinically stable may be allowed to participate at the discretion of the investigator.
Participants with alcohol consumption habits averaging > 210 g/week for men and >140 g/week for women in the past 1 month based on the answers to a drinking habits questionnaire.
Participants with uncontrolled diabetes (fasting blood glucose ≥ 180 mg/dL).
Participants with uncontrolled hypertension (blood pressure ≥ 160/100 mmHg).
Participants with a creatinine concentration ≥ 2 times the upper limit of site normal at V1.
Participants with AST (GOT) or ALT (GPT) ≥ 3 times the upper limit of site normal at V1.
Participation in a human application study or clinical trial less than 3 months prior.
Participants who were pregnant, lactating, or planning to become pregnant during the study period.
Participants with an allergic reaction to any of the foods in the investigational product.

ALT, alanine aminotransferase; AST, aspartate aminotransferase; NDI-K, Korean version of the Nepean Dyspepsia Index; V1, visit 1.

**Table 2:** Participant characteristics by group.

		FGK (*n* = 48)	Placebo (*n* = 45)
Sex, *n* (%)	*n*	48	45
	Male	12 (25.00)	7 (15.56)
	Female	36 (75.00)	38 (84.44)
	*p*-value ^1^	0.2589 (C)	

Age (year)	Mean ± SD	45.17 ± 10.53	40.67 ± 11.74
	Median	47.00	43.00
	(Min, Max)	(24.00, 68.00)	(20.00, 67.00)
	*p*-value ^2^	0.0545 (T)	

Height (cm)	Mean ± SD	162.95 ± 8.52	162.48 ± 8.38
	Median	162.15	160.70
	(Min, Max)	(147.00, 180.30)	(152.80, 186.50)
	*p*-value ^2^	0.4307 (W)	

Weight (kg)	Mean ± SD	64.36 ± 11.30	61.06 ± 13.63
	Median	62.70	56.50
	(Min, Max)	(42.90, 89.40)	(41.10, 112.00)
	*p*-value ^2^	0.0522 (W)	

Body mass index (kg/m^2^)	Mean ± SD	24.11 ± 2.97	23.02 ± 4.06
	Median	23.65	21.60
	(Min, Max)	(19.00, 33.90)	(17.00, 36.40)
	*p*-value ^2^	0.0136 (W)	

^1^ Comparison between groups using the chi-square test (C). ^2^ Comparison between groups using the two-sample *t*-test (T) or Wilcoxon’s rank-sum test (W). FGK, fermented gold kiwi; SD, standard deviation.

**Table 3:** Changes in GSRS epigastric symptoms scores and NDI-K score from baseline (V1) to week 8 (V5) in the Per Protocol Set.

Visit		GSRS Epigastric Symptoms Score		NDI-K Score	
		FGK (*n* = 48)	Placebo (*n* = 45)	FGK (*n* = 48)	Placebo (*n* = 45)
V1	Mean ± SD	25.10 ± 7.01	26.13 ± 7.13	72.92 ± 22.85	72.51 ± 24.67
	Median	25.50	26.00	73.00	72.00
	(Min, Max)	(11.00, 45.00)	(12.00, 43.00)	(34.00, 138.00)	(29.00, 139.00)
	*p*-value ^1^	0.4229 (W)		0.9346 (T)	
V5	Mean ± SD	13.65 ± 6.70	18.49 ± 9.66	25.06 ± 22.63	40.36 ± 34.17
	Median	11.50	15.00	18.00	29.00
	(Min, Max)	(8.00, 45.00)	(8.00, 49.00)	(0.00, 118.00)	(0.00, 146.00)
	*p*-value ^1^	0.0052 (W)		0.0364 (W)	
Change from V1 to V5	Mean ± SD	−11.46 ± 7.89	−7.64 ± 8.66	−47.85 ± 29.77	−32.16 ± 30.88
	Median	−11.50	−7.00	−47.50	−34.00
	(Min, Max)	(−32.00, 4.00)	(−28.00, 20.00)	(−118.00, 44.00)	(−89.00, 48.00)
	LS Mean ± SE ^§^	−11.71 ± 1.09	−7.38 ± 1.12	−47.74 ± 3.92	−32.28 ± 4.05
	*p*-value ^2^	<0.0001 (T)	<0.0001 (T)	<0.0001 (T)	<0.0001 (T)
Treatment-induced difference in change from V1 to V5 (treatment–placebo)	Mean ± SD	−3.81 ± 8.27		−15.70 ± 30.31	
	(95% CI ^†^)	(−7.22, −0.40)		(−28.19, −3.21)	
	LS mean difference ^§^	−4.33		−15.46	
	(95% CI ^‡^)	(−7.44, −1.22)		(−26.66, −4.27)	
	*p*-value ^3^	0.0069 (A)		0.0073 (A)	
	*p*-value ^4^	0.0083		0.0061	

^1^ Comparison between groups using the two-sample t-test (T) or Wilcoxon’s rank-sum test (W). ^2^ Comparison within groups using the paired *t*-test (T). ^3^ Comparison between groups using ANCOVA adjusted for baseline (A). ^4^ Comparison between groups using repeated measure ANOVA; *p*-value for interaction (time × treatment). ^§^ ANCOVA results adjusted for baseline. ^†^ 95% two-sided confidence interval for the difference of mean. ^‡^ 95% two-sided confidence interval for the difference of LS-mean. CI, confidence interval; FGK, fermented gold kiwi; GSRS, Gastrointestinal Symptom Rating Scale; NDI-K, Korean version of the Nepean Dyspepsia Index; SD, standard deviation; SE, standard error; V1, visit 1; V5, visit 5.

## Data Availability

The data presented in this study are included in the article/Supplementary Materials. Further inquiries can be directed to the corresponding author.

## Supplementary Materials

The following supporting information can be downloaded at: https://www.mdpi.com/article/10.3390/nu16162670/s1, Table S1. Prohibited Medications (or Foods) and Treatments; Table S2. Raw materials and mixing ratio of the study intervention; Table S3. Change from baseline in the GSRS total and lower gastrointestinal scores from baseline to weeks 2, 4, and 8 (PPS); Table S4. Change from baseline in the GSRS total scores and Lower Gastrointestinal Symptoms Total Score at 2, 4, and 8 weeks (PPS); Table S5. Change from baseline in the total GSRS reflux and Diarrhoea Symptom Score at 2, 4, and 8 weeks (PPS); Table S6. Changes in the GSRS dyspepsia and diarrhoea symptom score from baseline to weeks 2, 4, and 8 (PPS); Table S7. Changes in the GSRS constipation symptom scores from baseline to weeks 2, 4, and 8 (PPS); Table S8. Change from baseline in the FD-QoL scores at 2, 4, and 8 weeks (PPS); Table S9. Change from baseline in the FD-QoL eating domains and vitality scores at 2, 4, and 8 weeks (PPS); Table S10. Change from baseline in the FD-QoL Emotional Domain and Social Functioning scores at 2, 4, and 8 weeks (PPS); Table S11. Change in the TNF-α and IL-6 levels from baseline to 8 weeks (PPS); Table S12. Adverse events (SAS); Table S13. Haematological test results (SAS); Table S14. Blood chemistry test results (SAS); Table S15. Urinalysis results (SAS); Table S16. Vital signs (SAS); Table S17. Lifestyle (PPS); Table S18. 24-Hour recall dietary survey (PPS); Table S19. Abbreviations and glossary of terms.
