# Supplementary material for: Fermented Gold Kiwi for Improved Gastric Health: Evaluation of Efficacy and Safety in a Randomised, Double-Blind, Placebo-Controlled Trial
Source: Nutrients. 2024 Aug 13;16(16):2670. doi: 10.3390/nu16162670 (PMC11356964; doi:10.3390/nu16162670)
Supplement: Supplementary file 1 [file nutrients-16-02670-s001.zip › nutrients-3116197-supplementary.pdf]

Table S1. Prohibited Medications (or Foods) and Treatments

Medications for gastritis treatment	Proton pump inhibitors, gastrointestinal motility enhancers, H2 receptor antagonists, gastrin receptor antagonists, gastric mucosal protectants, probiotics, and other medications for gastritis treatment
Medications that can induce gastritis	Corticosteroids, NSAIDs, aspirin, etc. Use for analgesic and antipyretic purposes should not exceed 7 days during the test period
Traditional Korean medicine treatments	Acupuncture, herbal medicine, moxibustion, etc. For treating indigestion and gastritis
Health functional foods for stomach health	
Alcohol consumption	Men: >14 units/week, Women: >7 units/week

Table S2. Raw materials and mixing ratio of the study intervention

	Raw material	Mixing ratio (%)	Content (mg)
FGK	Gold Kiwi Lactobacillus ferment	25.00	5,000
	Purified water	74.10	14,820
	Gold Kiwi flavour	0.50	100
	Enzymatically processed stevia	0.20	40
	Compound *baicalensis* extract	0.20	40
	Total	**100.00**	**20,000**
Placebo	Pectin	1.20	240
	Oat flour	0.50	100
	Skim milk powder	0.60	120
	Citrus concentrate	3.00	600
	Gold Kiwi flavour	0.50	100
	DL-Apple acid	0.40	80
	Enzymatically processed stevia	0.20	40
	Complex goldenseal extract	0.20	40
	Malt extract	0.05	10

	Caramel colour	0.04	8
	Purified water	93.31	18,662
	Total	100.00	20.000

Table S3. Change from baseline in the GSRS total and lower GI scores at 2, 4, and 8 weeks (PPS)

		GSRS Total Score		NDI-K Score	
		FGK (N=48)	Placebo (N=45)	FGK (N=48)	Placebo (N=45)
Baseline (Visit 1)	Mean±SD	25.10±7.01	26.13±7.13	72.92±22.85	72.51±24.67
	Median	25.50	26.00	73.00	72.00
	(Min, Max)	(11.00, 45.00)	(12.00, 43.00)	(34.00, 138.00)	(29.00, 139.00)
	p-value[1]	0.4229 (W)		0.9346 (T)	
Week 2 (Visit 3)	Mean±SD	18.27±5.52	19.31±6.64	43.81±24.44	47.82±26.51
	Median	18.00	18.00	38.50	39.00
	(Min, Max)	(9.00, 34.00)	(9.00, 41.00)	(2.00, 107.00)	(8.00, 135.00)
	p-value[1]	0.6774 (W)		0.5641 (W)	
Change from Visit 1 to Visit 3	Mean±SD	-6.83±6.25	-6.82±5.75	-29.10±20.04	-24.69±23.30
	Median	-5.50	-6.00	-29.00	-19.00
	(Min, Max)	(-23.00, 3.00)	(-20.00, 3.00)	(-80.00, 17.00)	(-87.00, 29.00)
	LS Mean±SE§	-7.08±0.71	-6.56±0.74	-29.04±2.92	-24.76±3.01
	p-value[2]	<0.0001 (W)	<0.0001 (T)	<0.0001 (T)	<0.0001 (T)
Difference in change from Visit 1 to Visit 3 (treatment-placebo)	Mean±SD	-0.01±6.01		-4.42±21.68	
	(95% CI†)	(-2.49, 2.47)		(-13.35, 4.52)	
	LS mean difference§	-0.52		-4.28	
	(95% CI‡)	(-2.56, 1.53)		(-12.61, 4.06)	
	p-value[3]	0.7521 (W)		0.3106 (A)	

		GSRS Total Score		NDI-K Score	
		**FGK (N=48)**	**Placebo (N=45)**	**FGK (N=48)**	**Placebo (N=45)**
Week 4 (Visit 4)	Mean±SD	15.21±4.96	18.96±8.08	33.75±22.12	44.27±30.58
	Median	14.50	17.00	27.00	42.00
	(Min, Max)	(8.00, 29.00)	(8.00, 40.00)	(0.00, 91.00)	(0.00, 136.00)
	p-value[1]	0.0335 (W)		0.1397 (W)	
Change from Visit 1 to Visit 4	Mean±SD	-9.90±6.90	-7.18±6.85	-39.17±21.20	-28.24±25.16
	Median	-9.00	-7.00	-42.50	-26.00
	(Min, Max)	(-26.00, 1.00)	(-30.00, 7.00)	(-78.00, 43.00)	(-95.00, 24.00)
	LS Mean±SE§	-10.16±0.84	-6.90±0.87	-39.10±3.14	-28.32±3.24
	p-value[2]	<0.0001 (T)	<0.0001 (T)	<0.0001 (W)	<0.0001 (T)
Difference in change from Visit 1 to Visit 4 (treatment-placebo)	Mean±SD	-2.72±6.87		-10.92±23.20	
	(95% CI†)	(-5.55, 0.11)		(-20.48, -1.36)	
	LS mean difference§	-3.26		-10.78	
	(95% CI‡)	(-5.66, -0.87)		(-19.75, -1.81)	
	p-value[3]	0.0082 (A)		0.0056 (W)	
	p-value[4]	0.0083		0.0061	

	GSRS Total Score		NDI-K Score	
	**FGK (N=48)**	**Placebo (N=45)**	**FGK (N=48)**	**Placebo (N=45)**

Comparison between groups: p-value by two sample t-test (T) or Wilcoxon rank sum test (W)

[2] Comparison within groups: p-value by paired t-test (T) or Wilcoxon signed rank test (W)

[3] Comparison between groups: p-value by ANCOVA adjusted for baseline (A) or Wilcoxon rank sum test (W)

[4] Comparison between groups: p-value for interaction (time*treatment) by repeated measure ANOVA

§ ANCOVA results adjusted for baseline

† 95% two-sided confidence interval for the difference in mean

‡ 95% two-sided confidence interval for the difference in LS-mean

Table S4. Change from baseline in the GSRS total scores and Lower Gastrointestinal Symptoms Total Score at 2, 4, and 8 weeks (PPS)

		GSRS Total Score		Lower Gastrointestinal Symptoms Total Score	
		**FGK (N=48)**	**Placebo (N=45)**	**FGK (N=48)**	**Placebo (N=45)**
Baseline (Visit 1)	Mean±SD	46.90±12.16	48.53±11.90	21.79±7.10	22.40±6.94
	Median	44.50	48.00	21.00	23.00
	(Min, Max)	(29.00, 78.00)	(20.00, 74.00)	(9.00, 36.00)	(8.00, 36.00)
	p-value[1]	0.3099 (W)		0.6773 (T)	
Week 2 (Visit 3)	Mean±SD	34.83±9.96	36.04±12.82	16.56±5.59	16.73±7.11
	Median	35.00	32.00	16.00	14.00
	(Min, Max)	(17.00, 62.00)	(19.00, 72.00)	(7.00, 29.00)	(8.00, 36.00)
	p-value[1]	0.9877 (W)		0.7521 (W)	
Change from Visit 1 to Visit 3	Mean±SD	-12.06±10.90	-12.49±11.34	-5.23±6.53	-5.67±7.17
	Median	-9.00	-13.00	-4.00	-6.00
	(Min, Max)	(-42.00, 5.00)	(-42.00, 15.00)	(-23.00, 16.00)	(-25.00, 12.00)
	LS Mean±SE§	-12.44±1.38	-12.09±1.43	-5.40±0.81	-5.49±0.84
	p-value[2]	<0.0001 (W)	<0.0001 (T)	<0.0001 (T)	<0.0001 (T)
Difference in change from Visit 1 to Visit 3 (treatment-placebo)	Mean±SD	0.43±11.12		0.44±6.85	
	(95% CI†)	(-4.16, 5.01)		(-2.39, 3.26)	
	LS mean difference§	-0.35		0.09	
	(95% CI‡)	(-4.31, 3.61)		(-2.22, 2.41)	
	p-value[3]	0.6527 (W)		0.9357 (A)	
Week 4 (Visit 4)	Mean±SD	28.92±8.37	34.18±13.47	13.71±4.31	15.22±6.57
	Median	27.00	30.00	13.00	13.00

		GSRS Total Score		Lower Gastrointestinal Symptoms Total Score	
		FGK (N=48)	Placebo (N=45)	FGK (N=48)	Placebo (N=45)
	(Min, Max)	(15.00, 50.00)	(15.00, 67.00)	(7.00, 24.00)	(7.00, 30.00)
	p-value[1]	0.1325 (W)		0.6408 (W)	
Change from Visit 1 to Visit 4	Mean±SD	-17.98±11.55	-14.36±12.61	-8.08±6.59	-7.18±7.80
	Median	-17.00	-13.00	-8.00	-4.00
	(Min, Max)	(-45.00, 7.00)	(-59.00, 8.00)	(-24.00, 7.00)	(-29.00, 5.00)
	LS Mean±SE§	-18.44±1.44	-13.87±1.48	-8.29±0.75	-6.95±0.77
	p-value[2]	<0.0001 (T)	<0.0001 (W)	<0.0001 (T)	<0.0001 (W)
Difference in change from Visit 1 to Visit 4 (treatment-placebo)	Mean±SD	-3.62±12.08		-0.91±7.20	
	(95% CI†)	(-8.60, 1.35)		(-3.87, 2.06)	
	LS mean difference§	-4.57		-1.34	
	(95% CI‡)	(-8.68, -0.46)		(-3.48, 0.80)	
	p-value[3]	0.1113 (W)		0.3779 (W)	
Week 8 (Visit 5)	Mean±SD	25.98±12.64	34.09±17.12	12.33±6.23	15.60±8.20
	Median	23.00	27.00	11.00	12.00
	(Min, Max)	(15.00, 89.00)	(15.00, 91.00)	(7.00, 44.00)	(7.00, 42.00)
	p-value[1]	0.0091 (W)		0.0433 (W)	
Change from Visit 1 to Visit 5	Mean±SD	-20.92±14.15	-14.44±15.04	-9.46±7.72	-6.80±8.19
	Median	-21.00	-14.00	-10.00	-6.00
	(Min, Max)	(-53.00, 12.00)	(-54.00, 35.00)	(-26.00, 10.00)	(-26.00, 15.00)
	LS Mean±SE§	-21.28±1.96	-14.06±2.02	-9.64±0.97	-6.61±1.01

		GSRS Total Score		Lower Gastrointestinal Symptoms Total Score	
		**FGK (N=48)**	**Placebo (N=45)**	**FGK (N=48)**	**Placebo (N=45)**
	p-value[2]	<0.0001 (T)	<0.0001 (T)	<0.0001 (T)	<0.0001 (T)
Difference in change from Visit 1 to Visit 5 (treatment-placebo)	Mean±SD	-6.47±14.58		-2.66±7.95	
	(95% CI†)	(-12.48, -0.46)		(-5.94, 0.62)	
	LS mean difference§	-7.23		-3.03	
	(95% CI‡)	(-12.83, -1.62)		(-5.81, -0.24)	
	p-value[3]	0.0121 (A)		0.0333 (A)	
	p-value[4]	0.0145		0.1139	

Comparison between groups: p-value by two sample t-test (T) or Wilcoxon rank sum test (W)

[2] Comparison within groups: p-value by paired t-test (T) or Wilcoxon signed rank test (W)

[3] Comparison between groups: p-value by ANCOVA adjusted for baseline (A) or Wilcoxon rank sum test (W)

[4] Comparison between groups: p-value for interaction (time*treatment) by repeated measure ANOVA

§ ANCOVA results adjusted for baseline

† 95% two-sided confidence interval for the difference in mean

‡ 95% two-sided confidence interval for the difference in LS mean

Table S5. Change from baseline in the total GSRS reflux and Diarrhoea Symptom Score at 2, 4, and 8 weeks (PPS)

		Total GSRS reflux		Diarrhoea Symptom Score	
		**FGK (N=48)**	**Placebo (N=45)**	**FGK (N=48)**	**Placebo (N=45)**
(Visit 1)	Mean±SD	3.04±1.25	2.97±1.21	2.82±1.14	2.82±1.16
	Median	3.00	3.00	2.67	3.00
	(Min, Max)	(1.00, 5.50)	(1.00, 6.00)	(1.00, 5.33)	(1.00, 5.33)
	p-value[1]	0.7699 (T)		0.9927 (T)	
(Visit 3)	Mean±SD	2.13±0.90	2.12±1.02	2.26±0.97	2.19±1.10
	Median	2.00	2.00	2.00	1.67
	(Min, Max)	(1.00, 4.50)	(1.00, 5.50)	(1.00, 4.67)	(1.00, 4.67)
	p-value[1]	0.7655 (W)		0.4549 (W)	
to Visit 3	Mean±SD	-0.92±1.11	-0.84±1.02	-0.56±0.90	-0.63±1.01
	Median	-0.50	-0.50	-0.34	-0.34
	(Min, Max)	(-4.00, 2.00)	(-3.00, 1.00)	(-2.33, 3.00)	(-2.67, 2.00)
	LS Mean±SE§	-0.90±0.12	-0.87±0.12	-0.56±0.12	-0.63±0.12
	p-value[2]	<0.0001 (W)	<0.0001 (W)	<0.0001 (W)	0.0001 (T)
Difference in change from Visit 1 to Visit 3 (treatment-placebo)	Mean±SD	-0.07±1.07		0.07±0.95	
	(95% CI†)	(-0.51, 0.37)		(-0.33, 0.46)	
	LS mean difference§	-0.03		0.07	
	(95% CI‡)	(-0.36, 0.30)		(-0.27, 0.40)	
	p-value[3]	0.7258 (W)		0.9293 (W)	
(Visit 4)	Mean±SD	1.69±0.58	2.21±1.11	1.78±0.76	1.91±0.87
	Median	1.50	2.00	1.67	1.67

		Total GSRS reflux		Diarrhoea Symptom Score	
		**FGK (N=48)**	**Placebo (N=45)**	**FGK (N=48)**	**Placebo (N=45)**
	(Min, Max)	(1.00, 4.00)	(1.00, 5.00)	(1.00, 4.00)	(1.00, 3.67)
	p-value[1]	0.0466 (W)		0.6592 (W)	
to Visit 4	Mean±SD	-1.35±1.21	-0.76±1.05	-1.04±0.85	-0.91±1.07
	Median	-1.25	-0.50	-1.00	-0.34
	(Min, Max)	(-4.00, 0.50)	(-3.00, 1.50)	(-2.67, 0.67)	(-3.00, 0.67)
	LS Mean±SE§	-1.33±0.11	-0.78±0.12	-1.04±0.10	-0.91±0.10
	p-value[2]	<0.0001 (T)	<0.0001 (W)	<0.0001 (T)	<0.0001 (W)
Difference in change from Visit 1 to Visit 4 (treatment-placebo)	Mean±SD	-0.60±1.13		-0.12±0.96	
	(95% CI†)	(-1.07, -0.13)		(-0.52, 0.27)	
	LS mean difference§	-0.55		-0.13	
	(95% CI‡)	(-0.87, -0.23)		(-0.40, 0.15)	
	p-value[3]	0.0235 (W)		0.2343 (W)	
(Visit 5)	Mean±SD	1.45±0.84	2.17±1.35	1.58±0.94	2.04±1.24
	Median	1.00	1.50	1.33	1.67
	(Min, Max)	(1.00, 6.00)	(1.00, 6.50)	(1.00, 7.00)	(1.00, 6.33)
	p-value[1]	0.0010 (W)		0.1106 (W)	
to Visit 5	Mean±SD	-1.59±1.31	-0.80±1.07	-1.24±1.05	-0.78±1.19
	Median	-1.50	-0.50	-1.00	-0.66
	(Min, Max)	(-4.00, 1.00)	(-2.50, 2.00)	(-3.33, 1.67)	(-3.33, 2.00)
	LS Mean±SE§	-1.57±0.14	-0.82±0.15	-1.24±0.14	-0.78±0.14
	p-value[2]	<0.0001 (W)	<0.0001 (W)	<0.0001 (T)	<0.0001 (T)

		Total GSRS reflux		Diarrhoea Symptom Score	
		**FGK (N=48)**	**Placebo (N=45)**	**FGK (N=48)**	**Placebo (N=45)**
Difference in change from Visit 1 to Visit 5 (treatment-placebo)	Mean±SD	-0.79±1.20		-0.45±1.12	
	(95% CI†)	(-1.29, -0.30)		(-0.91, 0.01)	
	LS mean difference§	-0.75		-0.45	
	(95% CI‡)	(-1.16, -0.35)		(-0.85, -0.06)	
	p-value[3]	0.0045 (W)		0.0240 (A)	
	p-value[4]	0.0003		0.0477	

[1] Comparison between groups: p-value by two sample t-test (T) or Wilcoxon rank sum test (W)

[2] Comparison within groups: p-value by paired t-test (T) or Wilcoxon signed rank test (W)

[3] Comparison between groups: p-value by ANCOVA adjusted for baseline (A) or Wilcoxon rank sum test (W)

[4] Comparison between groups: p-value for interaction (time*treatment) by repeated measure ANOVA

§ ANCOVA results adjusted for baseline

† 95% two-sided confidence interval for the difference in mean

‡ 95% two-sided confidence interval for the difference in LS mean

Table S6. Change in the GSRS Dyspepsia and Diarrhoea Symptom Score at 2, 4, and 8 weeks from baseline (PPS)

		GSRS Dyspepsia		Abdominal Pain Score	
		**FGK (N=48)**	**Placebo (N=45)**	**FGK (N=48)**	**Placebo (N=45)**
(Visit 1)	Mean±SD	3.65±0.94	3.79±1.04	2.67	3.00
	Median	3.50	3.75	(1.33, 5.67)	(1.00, 5.00)
	(Min, Max)	(2.00, 6.00)	(1.00, 6.50)	0.1902 (W)	
	p-value[1]	0.3439 (W)			
(Visit 3)	Mean±SD	2.76±0.76	2.81±0.91	2.00±0.81	2.25±0.88
	Median	2.75	2.50	2.00	2.00
	(Min, Max)	(1.00, 4.25)	(1.50, 5.00)	(1.00, 4.67)	(1.00, 5.00)
	p-value[1]	0.9016 (W)		0.1506 (W)	
to Visit 3	Mean±SD	-0.89±0.85	-0.98±1.02	-0.85±0.94	-0.81±0.88
	Median	-0.75	-1.00	-0.67	-0.67
	(Min, Max)	(-3.00, 0.50)	(-3.25, 1.25)	(-3.67, 1.00)	(-3.00, 1.00)
	LS Mean±SE§	-0.93±0.11	-0.93±0.11	-0.91±0.10	-0.75±0.11
	p-value[2]	<0.0001 (W)	<0.0001 (T)	<0.0001 (W)	<0.0001 (T)
Difference in change from Visit 1 to Visit 3 (treatment-placebo)	Mean±SD	0.09±0.93		-0.05±0.91	
	(95% CI†)	(-0.30, 0.47)		(-0.42, 0.33)	
	LS mean difference§	0.00		-0.16	
	(95% CI‡)	(-0.30, 0.31)		(-0.46, 0.13)	
	p-value[3]	0.6159 (W)		0.8984 (W)	
(Visit 4)	Mean±SD	2.31±0.81	2.77±1.12	1.68±0.74	2.08±0.93
	Median	2.13	2.50	1.50	1.67

		GSRS Dyspepsia		Abdominal Pain Score	
		**FGK (N=48)**	**Placebo (N=45)**	**FGK (N=48)**	**Placebo (N=45)**
	(Min, Max)	(1.00, 4.25)	(1.00, 5.00)	(1.00, 5.00)	(1.00, 5.00)
	p-value[1]	0.0815 (W)		0.0206 (W)	
to Visit 4	Mean±SD	-1.34±0.83	-1.02±1.20	-1.17±0.99	-0.98±1.01
	Median	-1.50	-1.00	-1.00	-0.67
	(Min, Max)	(-3.00, 0.25)	(-5.50, 1.50)	(-4.00, 1.33)	(-3.33, 0.66)
	LS Mean±SE§	-1.38±0.13	-0.98±0.13	-1.24±0.11	-0.91±0.11
	p-value[2]	<0.0001 (T)	<0.0001 (W)	<0.0001 (T)	<0.0001 (W)
Difference in change from Visit 1 to Visit 4 (treatment-placebo)	Mean±SD	-0.32±1.03		-0.20±1.00	
	(95% CI†)	(-0.74, 0.11)		(-0.61, 0.22)	
	LS mean difference§	-0.40		-0.33	
	(95% CI‡)	(-0.76, -0.03)		(-0.64, -0.01)	
	p-value[3]	0.1078 (W)		0.1973 (W)	
(Visit 5)	Mean±SD	2.14±1.05	2.67±1.33	1.52±0.86	2.08±1.13
	Median	1.75	2.25	1.33	1.67
	(Min, Max)	(1.00, 5.75)	(1.00, 6.25)	(1.00, 5.33)	(1.00, 5.67)
	p-value[1]	0.0634 (W)		0.0011 (W)	
to Visit 5	Mean±SD	-1.51±1.14	-1.12±1.38	-1.33±1.07	-0.98±1.25
	Median	-1.63	-1.00	-1.17	-0.67
	(Min, Max)	(-3.75, 1.00)	(-5.00, 2.25)	(-4.34, 0.66)	(-3.33, 2.34)
	LS Mean±SE§	-1.55±0.16	-1.08±0.17	-1.40±0.14	-0.91±0.14
	p-value[2]	<0.0001 (T)	<0.0001 (T)	<0.0001 (W)	<0.0001 (T)

		GSRS Dyspepsia		Abdominal Pain Score	
		**FGK (N=48)**	**Placebo (N=45)**	**FGK (N=48)**	**Placebo (N=45)**
Difference in change from Visit 1 to Visit 5 (treatment-placebo)	Mean±SD	-0.38±1.26		-0.36±1.16	
	(95% CI†)	(-0.90, 0.14)		(-0.83, 0.12)	
	LS mean difference§	-0.47		-0.49	
	(95% CI‡)	(-0.93, 0.00)		(-0.88, -0.10)	
	p-value[3]	0.0501 (A)		0.1949 (W)	
	p-value[4]	0.0480		0.2546	

[1] Comparison between groups: p-value by two sample t-test (T) or Wilcoxon rank sum test (W)

[2] Comparison within groups: p-value by paired t-test (T) or Wilcoxon signed rank test (W)

[3] Comparison between groups: p-
value by ANCOVA adjusted for baseline (A) or Wilcoxon rank sum test(W)

[4] Comparison between groups: p-value for interaction (time*treatment) by repeated measure ANOVA

§ ANCOVA results adjusted for baseline

† 95% two-sided confidence interval for the difference in mean

‡ 95% two-sided confidence interval for the difference in LS mean

Table S71. Change in the GSRS constipation symptom score at 2, 4, and 8 weeks from baseline (PPS)

		FGK (N=48)	Placebo (N=45)
(Visit 1)	Mean±SD	3.07±1.32	3.27±1.53
	Median	3.00	3.00
	(Min, Max)	(1.00, 6.33)	(1.00, 7.00)
	p-value[1]	0.5071 (T)	
(Visit 3)	Mean±SD	2.26±1.11	2.41±1.22
	Median	2.00	2.00
	(Min, Max)	(1.00, 6.33)	(1.00, 6.33)
	p-value[1]	0.6617 (W)	
to Visit 3	Mean±SD	-0.81±1.20	-0.86±1.47
	Median	-0.34	-0.34
	(Min, Max)	(-4.00, 2.00)	(-5.67, 1.66)
	LS Mean±SE§	-0.86±0.15	-0.80±0.15
	p-value[2]	<0.0001 (W)	0.0005 (W)
Difference in change from Visit 1 to Visit 3 (treatment-placebo)	Mean±SD	0.05±1.34	
	(95% CI†)	(-0.50, 0.60)	
	LS mean difference§	-0.07	
	(95% CI‡)	(-0.49, 0.36)	
	p-value[3]	0.8141 (W)	
(Visit 4)	Mean±SD	1.97±0.99	2.24±1.28
	Median	1.67	2.00
	(Min, Max)	(1.00, 5.67)	(1.00, 6.67)

		FGK (N=48)	Placebo (N=45)
	p-value[1]	0.4839 (W)	
to Visit 4	Mean±SD	-1.10±1.25	-1.03±1.59
	Median	-1.00	-0.34
	(Min, Max)	(-4.33, 2.67)	(-6.00, 1.34)
	LS Mean±SE§	-1.16±0.15	-0.96±0.16
	p-value[2]	<0.0001 (T)	<0.0001 (W)
Difference in change from Visit 1 to Visit 4 (treatment-placebo)	Mean±SD	-0.07±1.43	
	(95% CI†)	(-0.66, 0.52)	
	LS mean difference§	-0.20	
	(95% CI‡)	(-0.64, 0.23)	
	p-value[3]	0.3254 (W)	
(Visit 5)	Mean±SD	1.74±1.14	2.24±1.31
	Median	1.33	1.67
	(Min, Max)	(1.00, 5.67)	(1.00, 5.67)
	p-value[1]	0.0264 (W)	
to Visit 5	Mean±SD	-1.33±1.34	-1.02±1.72
	Median	-1.33	-0.67
	(Min, Max)	(-4.33, 2.00)	(-6.00, 3.00)
	LS Mean±SE§	-1.40±0.17	-0.95±0.17
	p-value[2]	<0.0001 (T)	<0.0001 (W)
Difference in change from Visit 1 to Visit 5	Mean±SD	-0.31±1.53	
	(95% CI†)	(-0.94, 0.32)	

		FGK (N=48)	Placebo (N=45)
(treatment-placebo)	LS mean difference§	-0.45	
	(95% CI‡)	(-0.93, 0.03)	
	p-value[3]	0.0955 (W)	
	p-value[4]	0.5068	

[1] Comparison between groups: p-value by two sample t-test (T) or Wilcoxon rank sum test (W)

[2] Comparison within groups: p-value by paired t-test (T) or Wilcoxon signed rank test (W)

[3] Comparison between groups: p-
value by ANCOVA adjusted for baseline (A) or Wilcoxon rank sum test (W)

[4] Comparison between groups: p-value for interaction (time*treatment) by repeated measure ANOVA

§ ANCOVA results adjusted for baseline

† 95% two-sided confidence interval for the difference in mean

‡ 95% two-sided confidence interval for the difference in LS mean

Table S8. Change from baseline in the FD-QoL scores at 2, 4, and 8 weeks (PPS)

		FGK (N=48)	Placebo (N=45)
(Visit 2)	Mean±SD	21.17±10.34	19.67±11.65
	Median	21.00	19.00
	(Min, Max)	(3.00, 45.00)	(2.00, 48.00)
	p-value[1]	0.5125 (T)	
(Visit 3)	Mean±SD	14.69±10.17	16.58±11.83
	Median	13.50	12.00
	(Min, Max)	(0.00, 42.00)	(0.00, 51.00)
	p-value[1]	0.4624 (W)	
to Visit 3	Mean±SD	-6.48±10.42	-3.09±6.40
	Median	-5.00	-2.00
	(Min, Max)	(-38.00, 23.00)	(-22.00, 11.00)
	LS Mean±SE§	-6.25±1.16	-3.33±1.20
	p-value[2]	<0.0001 (W)	0.0023 (T)
Difference in change from Visit 2 to Visit 3 (treatment-placebo)	Mean±SD	-3.39±8.71	
	(95% CI†)	(-6.98, 0.20)	
	LS mean difference§	-2.92	
	(95% CI‡)	(-6.25, 0.40)	
	p-value[3]	0.0680 (W)	
(Visit 4)	Mean±SD	11.00±9.60	15.62±12.39
	Median	9.00	11.00
	(Min, Max)	(0.00, 51.00)	(0.00, 49.00)

		FGK (N=48)	Placebo (N=45)
	p-value[1]	0.0687 (W)	
to Visit 4	Mean±SD	-10.17±13.54	-4.04±7.15
	Median	-9.00	-2.00
	(Min, Max)	(-45.00, 32.00)	(-24.00, 15.00)
	LS Mean±SE§	-9.81±1.38	-4.42±1.43
	p-value[2]	<0.0001 (W)	0.0004 (T)
Difference in change from Visit 2 to Visit 4 (treatment-placebo)	Mean±SD	-6.12±10.93	
	(95% CI†)	(-10.63, -1.62)	
	LS mean difference§	-5.39	
	(95% CI‡)	(-9.34, -1.44)	
	p-value[3]	0.0024 (W)	
(Visit 5)	Mean±SD	8.02±10.11	14.47±14.58
	Median	5.00	7.00
	(Min, Max)	(0.00, 49.00)	(0.00, 47.00)
	p-value[1]	0.0289 (W)	
to Visit 5	Mean±SD	-13.15±13.34	-5.20±8.47
	Median	-12.00	-5.00
	(Min, Max)	(-45.00, 30.00)	(-24.00, 13.00)
	LS Mean±SE§	-12.87±1.52	-5.49±1.57
	p-value[2]	<0.0001 (W)	<0.0001 (W)
Difference in change from Visit 2 to Visit 5	Mean±SD	-7.95±11.25	
	(95% CI†)	(-12.58, -3.31)	

		FGK (N=48)	Placebo (N=45)
(treatment-placebo)	LS mean difference§	-7.38	
	(95% CI‡)	(-11.72, -3.03)	
	p-value[3]	<0.0001 (W)	
	p-value[4]	0.0004	

[1] Comparison between groups: p-value by two sample t-test (T) or Wilcoxon rank sum test (W)

[2] Comparison within groups: p-value by paired t-test (T) or Wilcoxon signed rank test (W)

[3] Comparison between groups: p-value by ANCOVA adjusted for baseline (A) or Wilcoxon rank sum test (W)

[4] Comparison between groups: p-value for interaction (time*treatment) by repeated measure ANOVA

§ ANCOVA results adjusted for baseline

† 95% two-sided confidence interval for the difference in mean

‡ 95% two-sided confidence interval for the difference in LS mean

Table S9. Change from baseline in the FD-QoL eating domains and vitality scores at 2, 4, and 8 weeks (PPS)

		FD-QoL eating domains		Vitality scores	
		**FGK (N=48)**	**Placebo (N=45)**	**FGK (N=48)**	**Placebo (N=45)**
(Visit 2)	Mean±SD	5.81±3.13	5.18±3.16	6.79±3.24	7.00±3.38
	Median	5.00	5.00	7.00	7.00
	(Min, Max)	(1.00, 13.00)	(0.00, 12.00)	(0.00, 13.00)	(1.00, 14.00)
	p-value[1]	0.3538 (W)		0.7622 (T)	
(Visit 3)	Mean±SD	4.17±3.40	4.42±3.14	4.92±2.91	6.00±3.57
	Median	3.50	4.00	4.50	5.00
	(Min, Max)	(0.00, 12.00)	(0.00, 13.00)	(0.00, 13.00)	(0.00, 14.00)
	p-value[1]	0.6760 (W)		0.2274 (W)	
to Visit 3	Mean±SD	-1.65±3.68	-0.76±2.44	-1.88±3.27	-1.00±2.42
	Median	-1.00	0.00	-1.00	-1.00
	(Min, Max)	(-11.00, 9.00)	(-9.00, 4.00)	(-12.00, 5.00)	(-8.00, 3.00)
	LS Mean±SE§	-1.51±0.41	-0.90±0.42	-1.92±0.37	-0.96±0.39
	p-value[2]	0.0014 (W)	0.0638 (W)	0.0002 (T)	0.0095 (W)
Difference in change from Visit 2 to Visit 3 (treatment-placebo)	Mean±SD	-0.89±3.14		-0.88±2.89	
	(95% CI†)	(-2.19, 0.41)		(-2.07, 0.32)	
	LS mean difference§	-0.60		-0.96	
	(95% CI‡)	(-1.77, 0.56)		(-2.02, 0.11)	
	p-value[3]	0.2284 (W)		0.1463 (W)	
(Visit 4)	Mean±SD	2.98±3.28	4.11±3.28	4.10±2.86	5.51±3.86
	Median	2.00	4.00	4.00	4.00

		FD-QoL eating domains		Vitality scores	
		**FGK (N=48)**	**Placebo (N=45)**	**FGK (N=48)**	**Placebo (N=45)**
	(Min, Max)	(0.00, 15.00)	(0.00, 13.00)	(0.00, 13.00)	(0.00, 14.00)
	p-value[1]	0.0410 (W)		0.1336 (W)	
to Visit 4	Mean±SD	-2.83±4.23	-1.07±2.50	-2.69±3.88	-1.49±2.79
	Median	-2.00	0.00	-3.00	-1.00
	(Min, Max)	(-12.00, 9.00)	(-11.00, 2.00)	(-12.00, 7.00)	(-10.00, 5.00)
	LS Mean±SE§	-2.66±0.44	-1.26±0.45	-2.74±0.43	-1.43±0.44
	p-value[2]	<0.0001 (W)	0.0061 (W)	<0.0001 (T)	0.0009 (T)
Difference in change from Visit 2 to Visit 4 (treatment-placebo)	Mean±SD	-1.77±3.50		-1.20±3.40	
	(95% CI†)	(-3.21, -0.32)		(-2.60, 0.20)	
	LS mean difference§	-1.40		-1.30	
	(95% CI‡)	(-2.65, -0.15)		(-2.53, -0.08)	
	p-value[3]	0.0018 (W)		0.0375 (A)	
(Visit 5)	Mean±SD	1.81±2.94	3.80±4.20	3.42±3.46	4.89±3.91
	Median	1.00	2.00	3.00	4.00
	(Min, Max)	(0.00, 14.00)	(0.00, 18.00)	(0.00, 15.00)	(0.00, 14.00)
	p-value[1]	0.0050 (W)		0.0521 (W)	
to Visit 5	Mean±SD	-4.00±4.19	-1.38±2.87	-3.38±3.16	-2.11±3.44
	Median	-4.00	-1.00	-3.00	-2.00
	(Min, Max)	(-13.00, 10.00)	(-10.00, 7.00)	(-12.00, 4.00)	(-10.00, 5.00)
	LS Mean±SE§	-3.85±0.47	-1.54±0.49	-3.41±0.44	-2.07±0.46
	p-value[2]	<0.0001 (W)	0.0007 (W)	<0.0001 (T)	0.0002 (T)

		FD-QoL eating domains		Vitality scores	
		**FGK (N=48)**	**Placebo (N=45)**	**FGK (N=48)**	**Placebo (N=45)**
Difference in change from Visit 2 to Visit 5 (treatment-placebo)	Mean±SD	-2.62±3.61		-1.26±3.30	
	(95% CI†)	(-4.11, -1.13)		(-2.62, 0.10)	
	LS mean difference§	-2.30		-1.34	
	(95% CI‡)	(-3.66, -0.95)		(-2.61, -0.08)	
	p-value[3]	<0.0001 (W)		0.0379 (A)	
	p-value[4]	0.0005		0.1474	

[1] Comparison between groups: p-value by two sample t-test (T) or Wilcoxon rank sum test (W)

[2] Comparison within groups: p-value by paired t-test (T) or Wilcoxon signed rank test (W)

[3] Comparison between groups: p-
value by ANCOVA adjusted for baseline (A) or Wilcoxon rank sum test (W)

[4] Comparison between groups: p-value for interaction (time*treatment) by repeated measure ANOVA

§ ANCOVA results adjusted for baseline

† 95% two-sided confidence interval for the difference in mean

‡ 95% two-sided confidence interval for the difference in LS mean

Table S10. Change from baseline in the FD-QoL Emotional Domain and Social Functioning scores at 2, 4, and 8 weeks (PPS)

		FD-QoL Emotional Domain		Social Functioning scores	
		**FGK (N=48)**	**Placebo (N=45)**	**FGK (N=48)**	**Placebo (N=45)**
(Visit 2)	Mean±SD	4.79±3.74	3.98±3.89	3.77±3.46	3.51±4.08
	Median	4.00	3.00	3.00	1.00
	(Min, Max)	(0.00, 15.00)	(0.00, 18.00)	(0.00, 12.00)	(0.00, 15.00)
	p-value[1]	0.1911 (W)		0.4887 (W)	
(Visit 3)	Mean±SD	2.85±2.77	3.42±3.82	2.75±2.99	2.73±3.73
	Median	2.00	2.00	1.00	1.00
	(Min, Max)	(0.00, 10.00)	(0.00, 16.00)	(0.00, 9.00)	(0.00, 16.00)
	p-value[1]	0.8272 (W)		0.6415 (W)	
to Visit 3	Mean±SD	-1.94±3.36	-0.56±2.11	-1.02±2.83	-0.78±2.25
	Median	-1.00	0.00	0.00	0.00
	(Min, Max)	(-13.00, 6.00)	(-8.00, 5.00)	(-12.00, 6.00)	(-8.00, 4.00)
	LS Mean±SE§	-1.78±0.35	-0.72±0.36	-0.98±0.32	-0.82±0.34
	p-value[2]	<0.0001 (W)	0.0575 (W)	0.0072 (W)	0.0166 (W)
Difference in change	Mean±SD	-1.38±2.82		-0.24±2.57	
from Visit 2 to Visit 3	(95% CI†)	(-2.55, -0.22)		(-1.30, 0.81)	
(treatment-placebo)	LS mean difference§	-1.06		-0.16	
	(95% CI‡)	(-2.06, -0.06)		(-1.08, 0.77)	
	p-value[3]	0.0465 (W)		0.8403 (W)	
(Visit 4)	Mean±SD	1.94±2.68	3.36±3.69	1.98±3.00	2.64±3.65
	Median	1.00	2.00	1.00	1.00

		FD-QoL Emotional Domain		Social Functioning scores	
		**FGK (N=48)**	**Placebo (N=45)**	**FGK (N=48)**	**Placebo (N=45)**
	(Min, Max)	(0.00, 15.00)	(0.00, 13.00)	(0.00, 13.00)	(0.00, 14.00)
	p-value[1]	0.0627 (W)		0.4776 (W)	
to Visit 4	Mean±SD	-2.85±4.37	-0.62±2.39	-1.79±4.03	-0.87±2.34
	Median	-2.00	0.00	-1.50	0.00
	(Min, Max)	(-13.00, 12.00)	(-8.00, 6.00)	(-12.00, 13.00)	(-8.00, 5.00)
	LS Mean±SE§	-2.63±0.40	-0.87±0.42	-1.73±0.40	-0.93±0.41
	p-value[2]	<0.0001 (W)	0.0851 (W)	0.0003 (W)	0.0142 (W)
Difference in change from Visit 2 to Visit 4 (treatment-placebo)	Mean±SD	-2.23±3.55		-0.93±3.32	
	(95% CI†)	(-3.70, -0.77)		(-2.29, 0.44)	
	LS mean difference§	-1.76		-0.80	
	(95% CI‡)	(-2.92, -0.60)		(-1.93, 0.34)	
	p-value[3]	0.0006 (W)		0.0715 (W)	
(Visit 5)	Mean±SD	1.52±2.52	3.07±4.31	1.27±2.34	2.71±4.62
	Median	1.00	1.00	0.00	0.00
	(Min, Max)	(0.00, 13.00)	(0.00, 17.00)	(0.00, 11.00)	(0.00, 18.00)
	p-value[1]	0.1184 (W)		0.3618 (W)	
to Visit 5	Mean±SD	-3.27±4.28	-0.91±2.61	-2.50±3.76	-0.80±2.62
	Median	-2.50	0.00	-2.00	0.00
	(Min, Max)	(-13.00, 10.00)	(-7.00, 7.00)	(-11.00, 6.00)	(-8.00, 5.00)
	LS Mean±SE§	-3.07±0.43	-1.13±0.45	-2.45±0.42	-0.86±0.43
	p-value[2]	<0.0001 (W)	0.0104 (W)	<0.0001 (W)	0.0438 (W)

		FD-QoL Emotional Domain		Social Functioning scores	
		**FGK (N=48)**	**Placebo (N=45)**	**FGK (N=48)**	**Placebo (N=45)**
Difference in change from Visit 2 to Visit 5 (treatment-placebo)	Mean±SD	-2.36±3.57		-1.70±3.26	
	(95% CI†)	(-3.83, -0.89)		(-3.04, -0.36)	
	LS mean difference§	-1.94		-1.59	
	(95% CI‡)	(-3.18, -0.70)		(-2.78, -0.41)	
	p-value[3]	0.0005 (W)		0.0035 (W)	
	p-value[4]	0.0004		0.0198	

[1] Comparison between groups: p-value by two sample t-test (T) or Wilcoxon rank sum test (W)

[2] Comparison within groups: p-value by paired t-test (T) or Wilcoxon signed rank test (W)

[3] Comparison between groups: p-value by ANCOVA adjusted for baseline (A) or Wilcoxon rank sum test (W)

[4] Comparison between groups: p-value for interaction (time*treatment) by repeated measure ANOVA

§ ANCOVA results adjusted for baseline

† 95% two-sided confidence interval for the difference in mean

‡ 95% two-sided confidence interval for the difference in LS mean

Table S11. Change in the TNF-α and IL-6 levels from baseline to 8 weeks (PPS)

		TNF-α		IL-6	
		**FGK (N=48)**	**Placebo (N=45)**	**FGK (N=48)**	**Placebo (N=45)**
(Visit 1)	Mean±SD	0.69±0.20	0.63±0.14	1.98±2.84	1.16±0.91
	Median	0.66	0.61	0.98	0.85
	(Min, Max)	(0.40, 1.23)	(0.34, 0.95)	(0.49, 16.63)	(0.29, 5.49)
	p-value[1]	0.3082 (W)		0.0696 (W)	
(Visit 5)	Mean±SD	0.66±0.23	0.59±0.17	1.71±2.44	1.04±0.99
	Median	0.60	0.59	1.09	0.75
	(Min, Max)	(0.37, 1.45)	(0.28, 1.04)	(0.30, 16.64)	(0.32, 6.03)
	p-value[1]	0.2127 (W)		0.0012 (W)	
to Visit 5	Mean±SD	-0.02±0.14	-0.04±0.10	-0.27±1.44	-0.11±0.51
	Median	-0.02	-0.04	0.03	-0.06
	(Min, Max)	(-0.33, 0.33)	(-0.33, 0.21)	(-8.81, 1.05)	(-1.56, 0.93)
	LS Mean±SE§	-0.02±0.02	-0.04±0.02	-0.17±0.14	-0.22±0.15
	p-value[2]	0.2848 (T)	0.0101 (T)	0.7542 (W)	0.1420 (T)
Difference in change from Visit 1 to Visit 5 (treatment-placebo)	Mean±SD	0.02±0.12		-0.15±1.10	
	(95% CI†)	(-0.03, 0.07)		(-0.61, 0.30)	
	LS mean difference§	0.02		0.05	
	(95% CI‡)	(-0.03, 0.07)		(-0.36, 0.46)	
	p-value[3]	0.3660(A)		0.5616 (W)	

	TNF-α		IL-6	
	**FGK (N=48)**	**Placebo (N=45)**	**FGK (N=48)**	**Placebo (N=45)**

[1] Comparison between groups: p-value by two sample t-test (T) or Wilcoxon rank sum test (W)

[2] Comparison within groups: p-value by paired t-test (T) or Wilcoxon signed rank test (W)

[3] Comparison between groups: p-
value by ANCOVA adjusted for baseline (A) or Wilcoxon rank sum test (W)

[4] Comparison between groups: p-value for interaction (time*treatment) by repeated measure ANOVA

§ ANCOVA results adjusted for baseline

† 95% two-sided confidence interval for the difference in mean

‡ 95% two-sided confidence interval for the difference in LS mean

Table S12. Adverse events (SAS)

		FGK (N=50)	Placebo (N=48)
		n(%) [F]	n(%) [F]
Adverse events		8(16.00) [10]	10(20.83) [17]
	(95% CI†)	(5.84, 26.16)	(9.34, 32.32)
	p-value[1]	0.5368(C)	
Adverse drug events		0(0.00) [0]	0(0.00) [0]
	(95% CI†)	(0.00, 0.00)	(0.00, 0.00)
	p-value[1]	-	
Serious adverse event		0(0.00) [0]	0(0.00) [0]
	(95% CI†)	(0.00, 0.00)	(0.00, 0.00)

Note: [F]=frequency

[1] Comparison between groups; p-value by Chi-square test(C) or Fisher's exact test(F)

Table S13. Haematological test results (SAS)

			FGK (N=50)	Placebo (N=48)
WBC	Baseline (Visit 1)	Mean±SD	5.75±1.39	5.46±1.27
		Median	5.48	5.31
		(Min, Max)	(3.49, 8.75)	(3.12, 10.06)
	Week 8 (Visit 5)	Mean±SD	5.77±1.25	5.44±1.19
		Median	5.51	5.41
		(Min, Max)	(3.53, 9.57)	(3.51, 9.21)
	Change from Visit 1 to Visit 5	Mean±SD	0.02±0.89	-0.01±0.83
		Median	0.01	-0.08
		(Min, Max)	(-1.88, 2.02)	(-1.78, 1.76)
		p-value[1]	0.8464 (T)	0.9038 (T)
RBC	Baseline (Visit 1)	Mean±SD	4.89±0.43	4.79±0.49
		Median	4.87	4.69
		(Min, Max)	(3.93, 5.97)	(3.80, 6.27)
	Week 8 (Visit 5)	Mean±SD	4.91±0.43	4.80±0.48
		Median	4.93	4.74
		(Min, Max)	(4.16, 6.03)	(4.00, 6.27)
	Change from Visit 1 to Visit 5	Mean±SD	0.02±0.21	0.00±0.22
		Median	-0.01	0.04
		(Min, Max)	(-0.40, 0.61)	(-0.50, 0.56)
		p-value[1]	0.5002 (T)	0.9578 (T)

			FGK<br>(N=50)	Placebo<br>(N=48)
		p-value[2]	0.6707 (T)	
Haemoglobin (Hb)	Baseline<br>(Visit 1)	Mean±SD<br>Median<br>(Min, Max)	14.65±1.34<br>14.50<br>(11.70, 17.50)	14.40±1.64<br>14.60<br>(8.80, 18.30)
	Week 8<br>(Visit 5)	Mean±SD<br>Median<br>(Min, Max)	14.69±1.32<br>14.60<br>(11.40, 17.70)	14.33±1.59<br>14.35<br>(9.40, 18.20)
	Change from<br>Visit 1 to Visit 5	N<br>Mean±SD<br>Median<br>(Min, Max)<br>p-value[1]<br>p-value[2]	50<br>0.04±0.59<br>0.00<br>(-0.90, 1.70)<br>0.6330 (T)<br>0.3410 (T)	48<br>-0.08±0.60<br>0.00<br>(-1.60, 0.90)<br>0.3916 (T)
Haematocrit (Hct)	Baseline<br>(Visit 1)	Mean±SD<br>Median<br>(Min, Max)	44.49±6.63<br>43.55<br>(35.50, 83.60)	43.08±4.36<br>43.10<br>(29.10, 53.60)
	Week 8<br>(Visit 5)	Mean±SD<br>Median<br>(Min, Max)	44.05±3.59<br>44.05<br>(36.70, 52.40)	43.01±4.04<br>43.25<br>(32.00, 53.20)
	Change from<br>Visit 1 to Visit 5	Mean±SD<br>Median	-0.44±6.30<br>0.10	-0.08±1.80<br>0.15

			FGK (N=50)	Placebo (N=48)
		(Min, Max)	(-42.20, 6.40)	(-3.80, 4.10)
		p-value[1]	0.4202 (W)	0.7684 (T)
		p-value[2]	0.6013 (W)	
Platelets	Baseline (Visit 1)	Mean±SD	285.46±62.96	267.06±56.95
		Median	290.50	263.00
		(Min, Max)	(120.00, 415.00)	(157.00, 424.00)
	Week 8 (Visit 5)	Mean±SD	282.40±58.03	262.50±66.45
		Median	290.50	260.00
		(Min, Max)	(112.00, 409.00)	(60.00, 487.00)
	Change from Visit 1 to Visit 5	Mean±SD	-3.06±32.18	-4.56±32.46
		Median	-2.50	0.50
		(Min, Max)	(-122.00, 68.00)	(-146.00, 66.00)
		p-value[1]	0.5676 (W)	0.4030 (W)
		p-value[2]	0.7898 (W)	

[1] Comparison within groups; p-value by paired t-test (T) or Wilcoxon signed rank test (W)

[2] Comparison between groups; p-value by two sample t-test (T) or Wilcoxon rank sum test (W)

Table S14. Blood chemistry test results (SAS)

			FGK (N=50)	Placebo (N=48)
AST (GOT)	Baseline	Mean±SD	22.84±5.87	23.94±10.78

			FGK (N=50)	Placebo (N=48)
	(Visit 1)	Median	21.50	21.50
		(Min, Max)	(14.00, 39.00)	(14.00, 68.00)
	Week 8	Mean±SD	24.18±12.38	22.69±10.28
	(Visit 5)	Median	23.00	20.00
		(Min, Max)	(12.00, 99.00)	(13.00, 77.00)
	Change from	Mean±SD	1.34±10.78	-1.25±9.15
	Visit 1 to Visit 5	Median	-0.50	0.00
		(Min, Max)	(-19.00, 60.00)	(-49.00, 19.00)
		p-value[1]	0.9960 (W)	0.4897 (W)
		p-value[2]	0.6766 (W)	
ALT (GPT)	Baseline	Mean±SD	19.96±7.11	20.33±12.35
	(Visit 1)	Median	20.00	16.50
		(Min, Max)	(9.00, 42.00)	(8.00, 68.00)
	Week 8	Mean±SD	20.48±8.13	20.44±15.41
	(Visit 5)	Median	18.00	15.00
		(Min, Max)	(8.00, 41.00)	(9.00, 76.00)
	Change from	Mean±SD	0.52±6.53	0.10±9.70
	Visit 1 to Visit 5	Median	0.00	0.00
		(Min, Max)	(-15.00, 21.00)	(-20.00, 32.00)
		p-value[1]	0.5760 (T)	0.7913 (W)
		p-value[2]	0.7005 (W)	

			FGK (N=50)	Placebo (N=48)
γ-GT	Baseline	Mean±SD	22.70±20.32	17.31±13.26
	(Visit 1)	Median	16.00	13.00
		(Min, Max)	(8.00, 116.00)	(7.00, 92.00)
	Week 8	Mean±SD	20.90±14.58	16.88±11.79
	(Visit 5)	Median	16.50	13.00
		(Min, Max)	(8.00, 79.00)	(7.00, 72.00)
	Change from	Mean±SD	-1.80±9.07	-0.44±5.85
	Visit 1 to Visit 5	Median	0.00	0.00
		(Min, Max)	(-53.00, 12.00)	(-20.00, 18.00)
		p-value[1]	0.4877 (W)	0.5039 (W)
		p-value[2]	0.9743 (W)	
Total bilirubin	Baseline	Mean±SD	0.86±0.37	0.87±0.48
	(Visit 1)	Median	0.81	0.74
		(Min, Max)	(0.31, 2.66)	(0.38, 3.24)
	Mean±SD	0.85±0.31	0.86±0.43	48
	Median	0.77	0.75	
	(Min, Max)	(0.41, 2.21)	(0.36, 2.55)	
	Mean±SD	-0.00±0.28	-0.01±0.38	48
	Median	0.05	0.03	
	(Min, Max)	(-0.81, 0.55)	(-1.07, 1.09)	
	p-value[1]	0.9191 (T)	0.8311 (T)	

			FGK (N=50)	Placebo (N=48)
	p-value[2]	0.9092 (T)		
Total protein	Baseline	Mean±SD	7.30±0.41	7.32±0.44
	(Visit 1)	Median	7.30	7.25
		(Min, Max)	(5.90, 8.00)	(6.50, 8.30)
	Week 8	Mean±SD	7.27±0.34	7.26±0.36
	(Visit 5)	Median	7.30	7.30
		(Min, Max)	(6.40, 8.20)	(6.50, 7.90)
	Change from	Mean±SD	-0.03±0.39	-0.05±0.37
	Visit 1 to Visit 5	Median	0.00	-0.05
		(Min, Max)	(-1.00, 1.30)	(-0.80, 0.80)
		p-value[1]	0.5339 (W)	0.3383 (T)
		p-value[2]	0.7921 (W)	
Albumin	Baseline	Mean±SD	4.45±0.20	4.45±0.24
	(Visit 1)	Median	4.40	4.45
		(Min, Max)	(3.90, 4.80)	(4.00, 5.00)
	Week 8	Mean±SD	4.42±0.24	4.38±0.20
	(Visit 5)	Median	4.45	4.30
		(Min, Max)	(4.00, 5.00)	(3.90, 5.00)
	Change from	Mean±SD	-0.03±0.22	-0.07±0.20
	Visit 1 to Visit 5	Median	0.00	-0.10
		(Min, Max)	(-0.50, 0.50)	(-0.50, 0.40)

			FGK (N=50)	Placebo (N=48)
		p-value[1]	0.3023 (T)	0.0256 (T)
		p-value[2]	0.4139 (T)	
Total cholesterol	Baseline (Visit 1)	Mean±SD	197.38±39.79	196.94±32.44
		Median	193.00	192.00
		(Min, Max)	(116.00, 319.00)	(128.00, 274.00)
	Week 8 (Visit 5)	Mean±SD	194.92±35.68	191.27±30.61
		Median	202.50	188.00
		(Min, Max)	(107.00, 257.00)	(109.00, 260.00)
	Change from Visit 1 to Visit 5	Mean±SD	-2.46±25.76	-5.67±22.41
		Median	1.50	-7.00
		(Min, Max)	(-100.00, 51.00)	(-62.00, 67.00)
		p-value[1]	0.9507 (W)	0.0863 (T)
		p-value[2]	0.1345 (W)	
Triglyceride	Baseline (Visit 1)	Mean±SD	111.70±66.40	98.27±73.19
		Median	84.00	80.00
		(Min, Max)	(40.00, 306.00)	(30.00, 507.00)
	Week 8 (Visit 5)	Mean±SD	107.46±55.67	96.50±49.86
		Median	88.50	83.00
		(Min, Max)	(38.00, 313.00)	(42.00, 261.00)

			FGK (N=50)	Placebo (N=48)
	Change from Visit 1 to Visit 5	Mean±SD	-4.24±43.66	-1.77±47.84
		Median	2.50	3.00
		(Min, Max)	(-130.00, 95.00)	(-272.00, 64.00)
		p-value[1]	0.4955 (T)	0.6204 (W)
		p-value[2]	0.7223 (W)	
HDL cholesterol	Baseline (Visit 1)	Mean±SD	63.26±15.63	66.96±16.67
		Median	64.50	64.50
		(Min, Max)	(29.00, 100.00)	(30.00, 116.00)
	Week 8 (Visit 5)	Mean±SD	62.34±12.97	66.31±15.29
		Median	61.50	66.50
		(Min, Max)	(38.00, 94.00)	(37.00, 100.00)
	Change from Visit 1 to Visit 5	Mean±SD	-0.92±7.44	-0.65±8.29
		Median	-1.00	0.00
		(Min, Max)	(-25.00, 18.00)	(-21.00, 17.00)
		p-value[1]	0.3863 (T)	0.5919 (T)
		p-value[2]	0.8635 (T)	
LDL cholesterol	Baseline (Visit 1)	Mean±SD	123.78±37.34	122.08±32.17
		Median	119.50	116.50
		(Min, Max)	(53.00, 238.00)	(71.00, 214.00)
	Week 8 (Visit 5)	Mean±SD	124.36±34.61	117.42±30.11
		Median	124.50	113.00
		(Min, Max)	(46.00, 182.00)	(52.00, 189.00)

			FGK (N=50)	Placebo (N=48)
	Change from Visit 1 to Visit 5	Mean±SD	0.58±23.66	-4.67±20.12
		Median	4.00	-5.00
		(Min, Max)	(-94.00, 46.00)	(-63.00, 36.00)
		p-value[1]	0.4340 (W)	0.1359 (W)
		p-value[2]	0.0913 (W)	
BUN	Baseline (Visit 1)	Mean±SD	13.10±3.58	12.54±3.45
		Median	12.55	11.80
		(Min, Max)	(6.60, 23.40)	(6.90, 23.70)
	Week 8 (Visit 5)	Mean±SD	12.95±3.28	12.57±2.76
		Median	12.20	11.70
		(Min, Max)	(7.90, 23.60)	(6.50, 19.50)
	Change from Visit 1 to Visit 5	Mean±SD	-0.14±3.69	0.03±2.96
		Median	-0.15	0.35
		(Min, Max)	(-9.80, 7.90)	(-8.40, 5.90)
		p-value[1]	0.7838 (T)	0.9421 (T)
		p-value[2]	0.7966 (T)	
Creatinine (Cr)	Baseline (Visit 1)	Mean±SD	0.80±0.15	0.77±0.13
		Median	0.76	0.73
		(Min, Max)	(0.58, 1.20)	(0.59, 1.16)
	Week 8 (Visit 5)	Mean±SD	0.79±0.15	0.77±0.14
		Median	0.76	0.74

			FGK (N=50)	Placebo (N=48)
		(Min, Max)	(0.58, 1.34)	(0.54, 1.26)
	Change from Visit 1 to Visit 5	Mean±SD	-0.01±0.07	0.01±0.08
		Median	0.00	0.01
		(Min, Max)	(-0.21, 0.15)	(-0.26, 0.23)
		p-value[1]	0.3322 (T)	0.4275 (W)
		p-value[2]	0.2636 (W)	
Glucose	Baseline (Visit 1)	Mean±SD	90.76±12.82	89.63±10.16
		Median	91.00	91.50
		(Min, Max)	(71.00, 158.00)	(58.00, 115.00)
	Week 8 (Visit 5)	Mean±SD	89.20±15.05	87.17±8.02
		Median	87.00	88.00
		(Min, Max)	(73.00, 161.00)	(64.00, 103.00)
	Change from Visit 1 to Visit 5	Mean±SD	-1.56±10.82	-2.46±11.81
		Median	-3.00	-2.00
		(Min, Max)	(-22.00, 58.00)	(-30.00, 25.00)
		p-value[1]	0.0166 (W)	0.1557 (T)
		p-value[2]	0.9490 (W)	

[1] Comparison within groups; p-value by paired t-test (T) or Wilcoxon signed rank test (W)

[2] Comparison between groups; p-value by two sample t-test (T) or Wilcoxon rank sum test (W)

Table S152. Urinalysis results (SAS)

			Normal	Abnormal
		**Week 8/Baseline**	**N (%)**	**N (%)**
Specific gravity	FGK	Normal, n (%)	47 (94.00)	2 (4.00)
	(N=50)	Abnormal, n (%)	1 (2.00)	0 (0.00)
		p-value[1]	0.5637	
	Placebo	Normal, n (%)	46 (95.83)	2 (4.17)
	(N=48)	Abnormal, n (%)	0 (0.00)	0 (0.00)
		p-value[1]	-	
pH	FGK	Normal, n (%)	50 (100.0)	0 (0.00)
	(N=50)	Abnormal, n (%)	0 (0.00)	0 (0.00)
		p-value[1]	-	
	Placebo	Normal, n (%)	48 (100.0)	0 (0.00)
	(N=48)	Abnormal, n (%)	0 (0.00)	0 (0.00)
		p-value[1]	-	
Protein	FGK	Normal, n (%)	38 (76.00)	9 (18.00)
	(N=50)	Abnormal, n (%)	2 (4.00)	1 (2.00)
		p-value[1]	0.0348	
	Placebo	Normal, n (%)	35 (72.92)	6 (12.50)
	(N=48)	Abnormal, n (%)	6 (12.50)	1 (2.08)
		p-value[1]	1.0000	

		Week 8/Baseline	Normal<br>N (%)	Abnormal<br>N (%)
Glucose	FGK<br>(N=50)	Normal, n (%)	47 (94.00)	0 (0.00)
		Abnormal, n (%)	2 (4.00)	1 (2.00)
		p-value[1]	0.1573	
	Placebo<br>(N=48)	Normal, n (%)	48 (100.0)	0 (0.00)
		Abnormal, n (%)	0 (0.00)	0 (0.00)
		p-value[1]	-	
Blood (RBC)	FGK<br>(N=50)	Normal, n (%)	22 (44.00)	11 (22.00)
		Abnormal, n (%)	7 (14.00)	10 (20.00)
		p-value[1]	0.3458	
	Placebo<br>(N=48)	Normal, n (%)	24 (50.00)	6 (12.50)
		Abnormal, n (%)	6 (12.50)	12 (25.00)
		p-value[1]	1.0000	

[1] Comparison within groups; p-value by McNemar's test.

Table S163. Vital signs test results (SAS)

			FGK (N=50)	Placebo (N=48)
SBP	Baseline (Visit 2)	Mean±SD	120.08±13.03	119.25±13.71
		Median	119.00	119.00
		(Min, Max)	(93.00, 147.00)	(92.00, 146.00)
	Week 8 (Visit 5)	Mean±SD	120.46±11.83	116.29±13.02
		Median	121.00	116.00
		(Min, Max)	(93.00, 141.00)	(92.00, 142.00)
	Change from Visit 2 to Visit 5	Mean±SD	0.38±8.29	-2.96±11.57
		Median	0.00	-1.00
		(Min, Max)	(-22.00, 24.00)	(-29.00, 21.00)
		p-value[1]	0.7472 (T)	0.0829 (T)
		p-value[2]	0.1055 (T)	
DBP	Baseline (Visit 2)	Mean±SD	72.80±8.66	72.98±10.25
		Median	73.00	75.00
		(Min, Max)	(54.00, 89.00)	(50.00, 90.00)
	Week 8 (Visit 5)	Mean±SD	74.06±7.41	71.88±9.62
		Median	74.50	69.50
		(Min, Max)	(61.00, 87.00)	(54.00, 88.00)
	Change from Visit 2 to Visit 5	Mean±SD	1.26±8.56	-1.10±8.94
		Median	1.00	0.50
		(Min, Max)	(-15.00, 20.00)	(-25.00, 14.00)

			FGK (N=50)	Placebo (N=48)
		p-value[1]	0.3029 (T)	0.3964 (T)
		p-value[2]	0.1841 (T)	
Pulse	Baseline (Visit 2)	Mean±SD	76.16±9.59	76.96±8.56
		Median	75.00	75.00
		(Min, Max)	(60.00, 98.00)	(62.00, 97.00)
	Week 8 (Visit 5)	Mean±SD	75.20±9.86	76.08±8.21
		Median	75.50	74.00
		(Min, Max)	(60.00, 95.00)	(61.00, 93.00)
	Change from Visit 2 to Visit 5	Mean±SD	-0.96±9.58	-0.88±8.42
		Median	0.00	-0.50
		(Min, Max)	(-25.00, 18.00)	(-23.00, 14.00)
		p-value[1]	0.8675 (W)	0.4753 (T)
		p-value[2]	0.8560 (W)	
Body Temperature	Baseline (Visit 2)	Mean±SD	36.61±0.15	36.64±0.25
		Median	36.60	36.60
		(Min, Max)	(36.30, 37.00)	(36.30, 37.50)
	Week 8 (Visit 5)	Mean±SD	36.59±0.21	36.63±0.23
		Median	36.60	36.60
		(Min, Max)	(36.00, 37.10)	(36.20, 37.20)
	Change from Visit 2 to Visit 5	Mean±SD	-0.02±0.25	-0.02±0.32
		Median	0.00	0.00

		FGK (N=50)	Placebo (N=48)
	(Min, Max)	(-0.60, 0.50)	(-1.10, 0.60)
	p-value[1]	0.5315 (T)	0.7168 (T)
	p-value[2]	0.9259 (T)	

[1] Comparison within groups; p-value by paired t-test (T) or Wilcoxon signed rank test (W)

[2] Comparison between groups; p-value by two sample t-test (T) or Wilcoxon rank sum test (W)

Table S174. Lifestyle (PPS)

			FGK (N=48)	Placebo (N=45)
Baseline (Visit 2)	smoking, n (%)	n	48	45
		No	43 (89.58)	42 (93.33)
		Former smoker	2 (4.17)	0 (0.00)
		Current smoker	3 (6.25)	3 (6.67)
		p-value[1]	0.5984 (F)	
	Eating habit, n (%)	n	48	45
		Regular	33 (68.75)	30 (66.67)
		Irregular	15 (31.25)	15 (33.33)
		p-value[1]	0.8299 (C)	
	Average meal duration, n (%)	n	48	45
		10 minutes or less	8 (16.67)	5 (11.11)
		10 to 20 minutes	34 (70.83)	35 (77.78)
		20 minutes or more	6 (12.50)	5 (11.11)
		p-value[1]	0.7041 (C)	
	Number of overeats, n (%)	n	48	45
		Less than 3 times/week	35 (72.92)	33 (73.33)
		More than 3 times/week	13 (27.08)	12 (26.67)
		p-value[1]	0.9639 (C)	
	Stress awareness, n (%)	n	48	45

		FGK (N=48)	Placebo (N=45)
	None at all	4 (8.33)	3 (6.67)
	Some	40 (83.33)	32 (71.11)
	Many	4 (8.33)	10 (22.22)
	Very Many	0 (0.00)	0 (0.00)
	p-value[1]	0.2049 (F)	
Exercise habits, n (%)	N	48	45
	0 times/week	9 (18.75)	8 (17.78)
	1~2 times/week	16 (33.33)	15 (33.33)
	3~4 times/week	12 (25.00)	12 (26.67)
	5 or more times/week	4 (8.33)	4 (8.89)
	Daily	7 (14.58)	6 (13.33)
	p-value[1]	1.0000 (F)	
Drinking status, n (%)	n	48	45
	No	29 (60.42)	28 (62.22)
	Disconnected	0 (0.00)	0 (0.00)
	Current drinkers	19 (39.58)	17 (37.78)
	p-value[1]	0.8582 (C)	
Alcohol consumption (g/week))	N	19	17
	Mean±SD	42.16±37.80	49.16±35.04
	Median	26.80	39.30
	(Min, Max)	(11.80, 161.10)	(11.80, 127.00)
	p-value[2]	0.3802 (W)	

			FGK (N=48)	Placebo (N=45)
	Caffeine (coffee), n (%)	n	48	45
		Don't drink	7 (14.58)	5 (11.11)
		Drink	41 (85.42)	40 (88.89)
		p-value[1]	0.6177 (C)	
	Coffee intake (cups/week)	n	41	40
		Mean±SD	11.93±7.36	9.80±6.03
		Median	10.00	7.00
		(Min, Max)	(1.00, 35.00)	(1.00, 21.00)
		p-value[2]	0.2260 (W)	
	Caffeine (chocolate), n (%)	n	48	45
		Not eating	42 (87.50)	39 (86.67)
		Eat	6 (12.50)	6 (13.33)
		p-value[1]	0.9046 (C)	
	Chocolate intake (g/week)	n	6	6
		Mean±SD	46.83±34.39	73.67±54.47
		Median	42.00	51.00
		(Min, Max)	(10.00, 102.00)	(34.00, 170.00)
		p-value[2]	0.3316 (T)	
Week 8 (Visit 5)	Smoking status, n (%)	n	48	45
		No	45 (93.75)	42 (93.33)

		FGK (N=48)	Placebo (N=45)
	Former smokers	0 (0.00)	0 (0.00)
	Current smokers	3 (6.25)	3 (6.67)
	p-value[1]	1.0000 (F)	
Eating habits, n (%)	n	48	45
	Regular	39 (81.25)	36 (80.00)
	Irregular	9 (18.75)	9 (20.00)
	p-value[1]	0.8788 (C)	
Average meal duration, n (%)	n	48	45
	10 minutes or less	4 (8.33)	3 (6.67)
	10 to 20 minutes	36 (75.00)	38 (84.44)
	20 minutes or more	8 (16.67)	4 (8.89)
	p-value[1]	0.5546 (F)	
Number of overeats, n (%)	n	48	45
	Less than 3 times/week	40 (83.33)	40 (88.89)
	More than 3 times/week	8 (16.67)	5 (11.11)
	p-value[1]	0.4400 (C)	
Stress awareness, n (%)	n	48	45
	None at all	7 (14.58)	3 (6.67)
	Some	36 (75.00)	34 (75.56)
	Many	5 (10.42)	5 (11.11)
	Very Many	0 (0.00)	3 (6.67)

		FGK (N=48)	Placebo (N=45)
	p-value[1]	0.2526 (F)	
Exercise habits, n (%)	N	48	45
	0 times/week	11 (22.92)	8 (17.78)
	1~2 times/week	13 (27.08)	11 (24.44)
	3~4 times/week	16 (33.33)	17 (37.78)
	5 or more times/week	3 (6.25)	5 (11.11)
	Daily	5 (10.42)	4 (8.89)
	p-value[1]	0.8949 (F)	
Drinking status, n (%)	n	48	45
	No	29 (60.42)	23 (51.11)
	Disconnected	0 (0.00)	0 (0.00)
	Current drinkers	19 (39.58)	22 (48.89)
	p-value[1]	0.3664 (C)	
Alcohol consumption (g/week))	N	19	22
	Mean±SD	47.89±40.73	40.50±24.54
	Median (Min, Max)	39.30 (7.50, 161.10)	37.30 (11.80, 117.80)
	p-value[2]	0.9056 (W)	
Caffeine (coffee), n (%)	n	48	45
	Don’t drink	7 (14.58)	6 (13.33)
	Drink	41 (85.42)	39 (86.67)
	p-value[1]	0.8621 (C)	

		FGK (N=48)	Placebo (N=45)
Coffee intake (cups/week)	n	41	39
	Mean±SD	10.61±6.93	10.77±5.39
	Median	7.00	7.00
	(Min, Max)	(2.00, 40.00)	(3.00, 21.00)
	p-value[2]	0.5991 (W)	
Caffeine (chocolate), n (%)	n	48	45
	Not eating	41 (85.42)	42 (93.33)
	Eat	7 (14.58)	3 (6.67)
	p-value[1]	0.3186 (F)	
Chocolate intake (g/week)	n	7	3
	Mean±SD	70.86±123.49	51.67±18.01
	Median	34.00	51.00
	(Min, Max)	(10.00, 350.00)	(34.00, 70.00)
	p-value[2]	0.1944 (W)	

[1] Comparison between groups; p-value by Chi-square test (C) or Fisher's exact test (F)

[2] Comparison between groups; p-value by two sample t-test (T) or Wilcoxon rank sum test (W)

Table S18. 24-Hour Recall Dietary Survey (PPS)

		FGK (N=48)	Placebo (N=45)
Baseline (Visit 2)	n	48	45
	Mean±SD	1520.06±462.37	1493.91±456.95
	Median	1441.17	1449.45
	(Min, Max)	(702.92, 2595.33)	(677.87, 2656.70)
	p-value[1]	0.7846 (T)	
Week 8 (Visit 5)	n	48	45
	Mean±SD	1489.65±448.15	1377.40±556.98
	Median	1429.24	1438.59
	(Min, Max)	(628.57, 3061.82)	(594.58, 3248.16)
	p-value[1]	0.1925 (W)	
Change from Visit 2 to Visit 5	n	48	45
	Mean±SD	-30.41±481.77	-116.51±639.98
	Median	7.29	-198.38
	(Min, Max)	(-1320.13, 1092.15)	(-1431.40, 1448.46)
	p-value[1]	0.4637 (T)	

[1] Comparison between groups; p-value by two sample t-test (T) or Wilcoxon rank sum test (W)

Table S19. Abbreviations and glossary of terms

ADR	Adverse drug reaction
AE	Adverse event
ALP	Alkaline phosphatase
ALT	Alanine aminotransferase
ANCOVA	Analysis of covariance
ANOVA	Analysis of variance
AST	Aspartate aminotransferase
BUN	Blood urea nitrogen
CRP	C-reactive protein
DBP	Diastolic blood pressure
ESR	Erythrocyte sedimentation rate
FAS	Full analysis set
FD-QoL	functional dyspepsia-related quality of life
FGK	Fermented Gold Kiwi
GI	Gastrointestinal
GSRS	Gastrointestinal Symptom Rating Scale
γ-GT	Gamma-glutamyl transferase
HCG	Human chorionic gonadotropin
HDL-C	High density lipoprotein-cholesterol
IL-6	Interleukin-6
ITT	Intention to Treat
IPAQ	International Physical Activity Questionnaires
IRB	Institutional review board
IWRS	Interactive web response system

LDL-C	Low-density lipoprotein-cholesterol
MET	Metabolic equivalent of task
NDI-K	Korean version of the Nepan Dyspepsia Index
NSAIDs	Non-steroidal anti-inflammatory drugs
PGE2	Prostaglandin E2
PPS	Per protocol set
RBC	Red blood cell
SAE	Serious adverse event
SAS	Safety analysis set
SBP	Systolic blood pressure
TG	Triglyceride
TNF- α	Tumour Necrosis Factor-α
UNL	Upper normal limit
WBC	White blood cell
